# Cost-effectiveness of an insertable cardiac monitor in a high-risk population in the US

**DOI:** 10.1186/s12872-023-03073-6

**Published:** 2023-01-25

**Authors:** Mitchell S. V. Elkind, Klaus K. Witte, Scott E. Kasner, Laura M. Sawyer, Frank W. Grimsey Jones, Claudia Rinciog, Stelios Tsintzos, Sarah C. Rosemas, David Lanctin, Paul D. Ziegler, Matthew R. Reynolds

**Affiliations:** 1 Department of Neurology, Vagelos College of Physicians and Surgeons, and Department of Epidemiology, Mailman School of Public Health, Columbia University, New York, NY USA; 2 Leeds Institute for Cardiovascular and Metabolic Medicine, University of Leeds, Leeds, UK; 3 University Clinic, RWTH, Aachen, Germany; 4 Department of Neurology, University of Pennsylvania, Philadelphia, PA USA; 5 Symmetron Limited, 8 Devonshire Square, London, EC2M 4PL UK; 6 Medtronic, Mounds View, MN USA; 7 Baim Institute for Clinical Research, Boston, MA USA; 8 Lahey Hospital and Medical Center, Burlington, MA USA

**Keywords:** Atrial fibrillation, Cardiology, Stroke, Diagnostics, Economic evaluation

## Abstract

**Background:**

Insertable cardiac monitors (ICMs) are a clinically effective means of detecting atrial fibrillation (AF) in high-risk patients, and guiding the initiation of non-vitamin K oral anticoagulants (NOACs). Their cost-effectiveness from a US clinical payer perspective is not yet known. The objective of this study was to evaluate the cost-effectiveness of ICMs compared to standard of care (SoC) for detecting AF in patients at high risk of stroke (CHADS_2_ ≥ 2), in the US.

**Methods:**

Using patient data from the REVEAL AF trial (n = 393, average CHADS_2_ score = 2.9), a Markov model estimated the lifetime costs and benefits of detecting AF with an ICM or with SoC (specifically intermittent use of electrocardiograms and 24-h Holter monitors). Ischemic and hemorrhagic strokes, intra- and extra-cranial hemorrhages, and minor bleeds were modelled. Diagnostic and device costs, costs of treating stroke and bleeding events and medical therapy—specifically costs of NOACs were included. Costs and health outcomes, measured as quality-adjusted life years (QALYs), were discounted at 3% per annum, in line with standard practice in the US setting. One-way deterministic and probabilistic sensitivity analyses (PSA) were undertaken.

**Results:**

Lifetime per-patient cost for ICM was $31,116 versus $25,330 for SoC. ICMs generated a total of 7.75 QALYs versus 7.59 for SoC, with 34 fewer strokes projected per 1000 patients. The model estimates a number needed to treat of 29 per stroke avoided. The incremental cost-effectiveness ratio was $35,528 per QALY gained. ICMs were cost-effective in 75% of PSA simulations, using a $50,000 per QALY threshold, and a 100% probability of being cost-effective at a WTP threshold of $150,000 per QALY.

**Conclusions:**

The use of ICMs to identify AF in a high-risk population is likely to be cost-effective in the US healthcare setting.

**Supplementary Information:**

The online version contains supplementary material available at 10.1186/s12872-023-03073-6.

## Introduction

Atrial fibrillation (AF) is associated with an increased risk of stroke [1, 2]. Anticoagulation therapy particularly with non-vitamin-K oral anticoagulants (NOACs) is effective at reducing ischemic stroke (IS) risk in patients with AF [3]. This benefit only occurs in the presence of a clear electrocardiographic diagnosis of AF due to the increased risk of bleeding associated with NOACs: patients who do not have AF that receive face an increased risk of bleeding with no reduction in IS risk [4]. Because the symptoms associated with suspected AF can vary widely, and AF episodes themselves may be transient [5], there is a risk that diagnoses are missed by opportunistic and short-term monitoring solutions [such as intermittent 24-h Holter monitor or electrocardiogram (ECG) use].

Insertable cardiac monitors (ICMs) can provide continuous long-term cardiac monitoring in patients with potential arrhythmias. The REVEAL AF study [6] (ClinicalTrials.gov, Number: NCT01727297) monitored the heart rhythms of patients at high risk of AF and non-specific symptoms. Study participants had a CHADS2 score of at least 3 or a score of 2 with at least one additional stroke risk factor. All 393 patients had 24 h or more of external monitoring within 90 days prior to enrolment or ICM insertion, with any prior AF detection being an exclusion criterion. In 81 enrolled patients, the risk profile included a previous diagnosis of heart failure (HF). It is increasingly appreciated that AF and HF are co-morbidities, with a bidirectional adverse impact on patient outcomes [7].

While the REVEAL AF study demonstrated the ability of ICMs to detect previously undiagnosed AF, whether the use of ICMs for AF detection in a high-risk population is cost-effective is unknown. The objective of this analysis was to estimate the cost effectiveness of ICMs compared to standard of care in detecting AF, in a population at high risk for AF, from a US clinical payer perspective. A list of abbreviations used within this paper is presented in Table 1.

**Table 1:** List of abbreviations

Abbreviation	Definition
AF	Atrial fibrilation
CHADS_2_	congestive heart failure, hypertension, age > 75 years, diabetes mellitus, stroke/transient ischemia attack/ thromboembolism
CRNM	Clinically relevant non-major
ECG	Electrocardiogram
ECH	Extra cranial hemorrhage
HF	Heart failure
HS	Hemorrhagic stroke
ICER	Incremental cost-effectiveness ratio
ICH	Intracranial hemorrhage
ICM	Insertable cardiac monitor
IS	Ischemic stroke
NNT	Number needed to treat
NOAC	Non-vitamin K oral anticoagulant
PSA	Probabilistic sensitivity analysis
QALY	Quality-adjusted life year
QoL	Quality of life
SoC	Standard of care
US	United States

## Methods

A model was adapted based on a previous analysis in the UK setting [8], to a US setting. The model was a Markov model, which, as a cohort state transition model, was designed to simulate costs and benefits of monitoring for AF with an ICM compared to standard of care (SoC), in patients with a high risk of stroke and no confirmed AF diagnosis. It was developed using Excel, Microsoft 365. Costs of monitoring and treatment represent a US payer perspective.

Health benefits were expressed as quality-adjusted life years (QALYs) and discounted, along with costs, at 3% annually. The Markov model used a lifetime horizon to capture all costs and health-related quality of life (QoL) benefits. Life-years gained and IS events avoided were also compared between ICM and SoC strategies. The model cohort was set up to have the same baseline characteristics as recorded in the REVEAL AF study [6] (Additional file 1: Table S1), and the duration of model cycles (3 months) also matched the observation intervals in REVEAL AF.

### Model structure

**Fig. 1:**
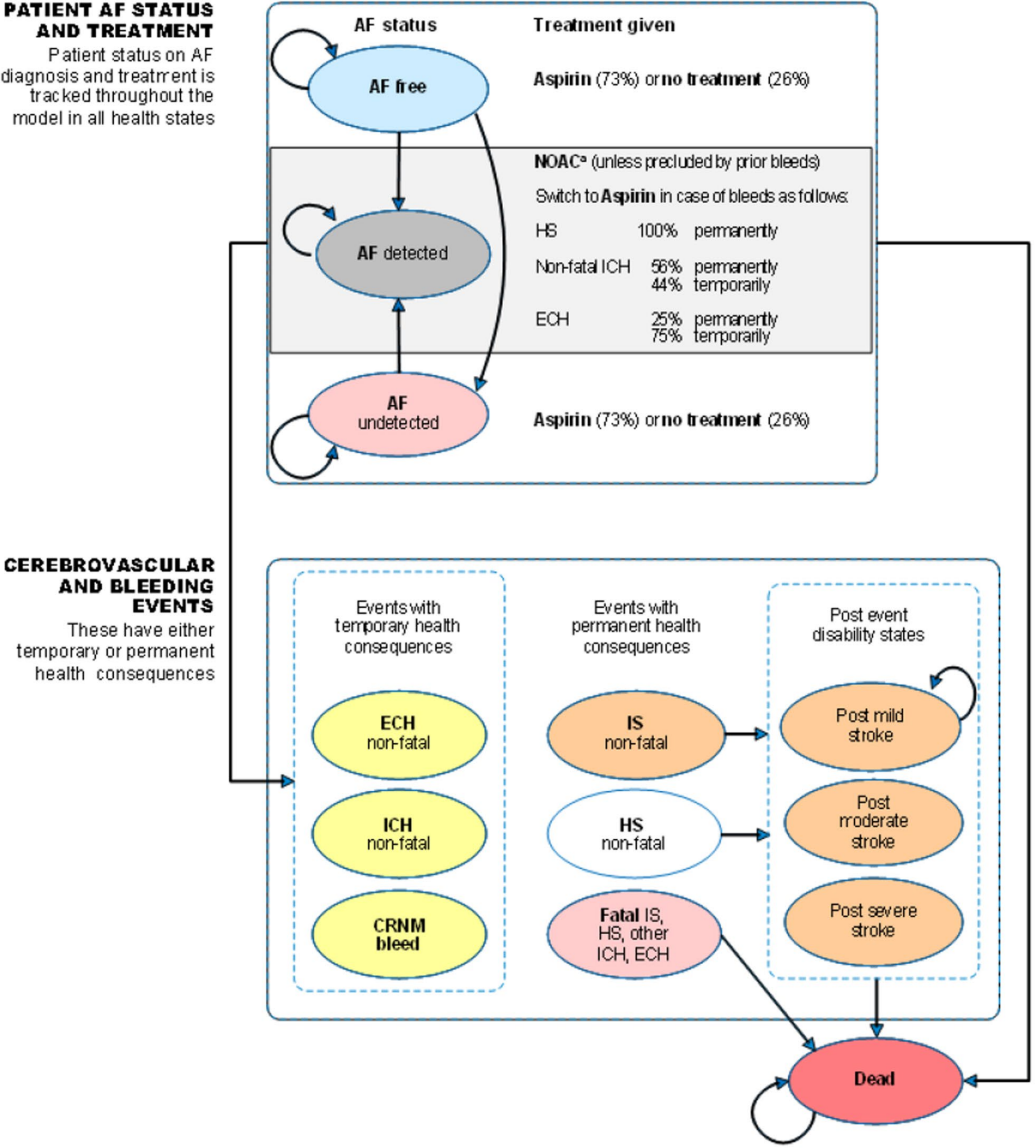
Model flow. *Notes*: a, NOACs are administered in base-case analysis, warfarin is substituted in sensitivity analysis. Abbreviations: AF: atrial fibrillation; CRNM: clinically relevant non-major; ECH: extracranial hemorrhage; HS: hemorrhagic stroke; ICH: intracranial hemorrhage; IS: ischemic stroke; NOAC: non-vitamin K oral anticoagulants

The model simulates the expected efficacy of ICM or SoC monitoring in detecting AF. If AF is detected, patients begin NOAC therapy, which reduces the risk of IS. The increased risk of major bleeds associated with NOACs is modelled, as is anticoagulant discontinuation. All relevant costs and health benefits are estimated separately for ICM and SoC. Movement of patients through the model is shown in Fig. 1.

The hypothetical cohort is assumed to enter the model without an established diagnosis of AF. In each cycle, their AF status can remain the same or change and be detected or go undetected. Prior to AF detection, patients receive either aspirin (73%) or no treatment (27%) [6]. When AF is detected, the patient is assumed to start NOAC therapy, which is associated with a reduction in the risk of IS but an increase in the risk of hemorrhagic strokes (HS) and other bleeding events. Following a stroke, patients move to one of three post-stroke health states where they have a reduced QoL and higher costs, corresponding to the severity of the stroke experienced. Stroke events can also be fatal. Detection of AF and initiation of NOAC therapy can still occur among patients in post-stroke health states.

### SoC and ICM clinical practice

SoC was assumed to involve intermittent 24-h Holter and ECG use (see Additional file 1: Sect. 8.2). ICM was assumed to involve the insertion and regular monitoring of an ICM, followed by device explantation after battery expiry at three years and a return to SoC for the remaining years.

### Population

The baseline characteristics of the patient cohort match the REVEAL AF trial population. Mean age was 71.3 years, 73% were on aspirin, and 20% had one or more strokes in the year prior to recruitment. The mean virtual CHADS_2_ score was 2.94 (Additional file 1: Sect. 1). CHADS_2_ is a clinical prediction scoring system to determine stroke risk in patients with atrial fibrillation. It accounts for history of congestive heart failure, hypertension, age (> 75 years), diabetes mellitus and stroke/transient ischemia attack/ thromboembolism [3].

### AF detection, frequency and management post-detection

**Fig. 2:**
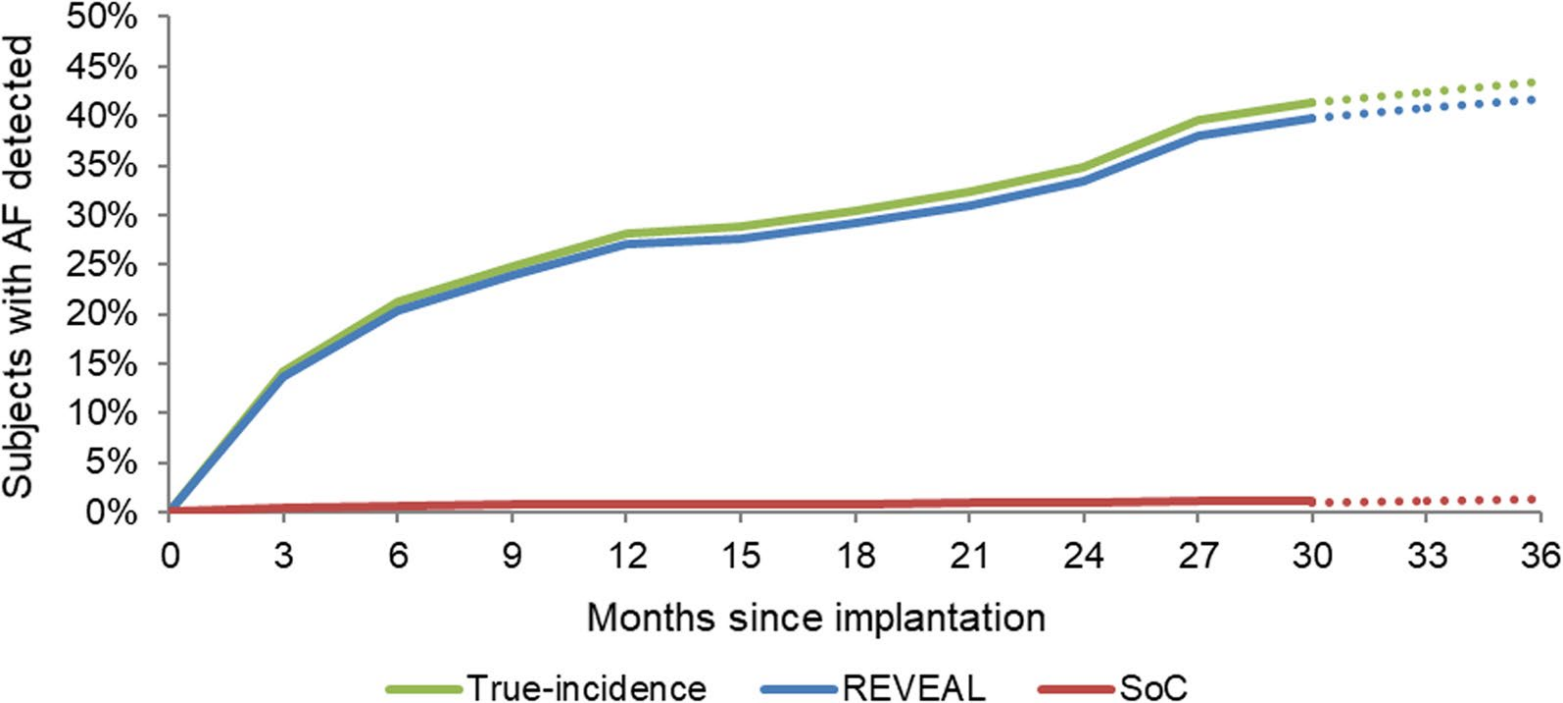
AF detection rates in ICM and SoC—all patients. *Notes*: Both ICM and SoC detection curves are modelled based on analysis of the REVEAL AF trial dataset (5). The AF burden is extrapolated from the end of the 30-month study and the end of the battery life at 36 months, using a linear extrapolation from month 3 to month 30 (3.9% per 3-month cycle). Abbreviations: AF: atrial fibrillation; ICM: insertable cardiac monitor; SoC, standard of care

AF detection rates for ICM were based on those seen in the REVEAL AF study [6]. Our base case analysis used the study primary endpoint definition of AF as an episode lasting ≥ 6 min. Given the lack of consensus around the duration of AF to both diagnose the condition and for the risk of stroke to increase, device recordings were re-analyzed to also create a scenario in which AF was only diagnosed if episodes lasted for ≥ 5.5 h [9]. The REVEAL AF trial data informed the rate of AF detection for the first 30 months in the model (Fig. 2). After 30 months, the AF risk was extrapolated by fitting a logarithmic curve (excluding the first 3 months). To reflect the potential for some AF episodes to go undetected by ICMs, the model assumed a device sensitivity of 96.1% [10].

For SoC, the rate of detection was sourced from a simulated comparison of AF monitoring strategies using trial data from REVEAL AF [11]. Using data from this comparison, the relative diagnostic yield of ICM compared to SoC, expressed as a hazard ratio, was 33.9 (95% CI 13.2—NE). Further details are provided in the Additional file 1: Table S4.

Modelled patients were given NOAC therapy in the same cycle they received an AF diagnosis. A scenario analysis explored the impact of a lower NOAC prescription rate (66.35%) post-AF diagnosis; this scenario was informed by prescription rate in the REVEAL AF study [6].

### Ischemic stroke risk and severity

Per 3-month cycle, the risk of ischemic stroke (IS) was based on AF status, virtual CHADS_2_ score, anticoagulation treatment and patient age (see Table 2).It was necessary to combine evidence from a number of publications to allow this risk stratification [1, 12–15]. The increased IS risk associated with device-detected AF was taken from Mahajan et al. [15]. IS severity was modelled using estimates from previous cost-effectiveness publications [16, 17]. The IS severity distribution was independent of patients’ current anticoagulation treatment (see Additional file 1: Table S12). An IS was assumed to have a permanent impact on a patient’s health management costs, QoL and mortality risk, with the level of impact corresponding to stroke severity (Additional file 1: Tables S15 and S17).

**Table 2:** Annual ischemic stroke probability and severity by CHADS2 risk score, AF status and anticoagulant treatment received

CHADS_2_ score	No AF		Subclinical AF			
	No treatment (%)	Aspirin (%)	No treatment (%)	Aspirin (%)	NOAC*^,†^ (%)	Warfarin* (%)
0^‡^	0.2	0.2	0.6	0.4	0.2	0.1
1^‡^	0.6	0.5	1.6	1.1	0.4	0.4
2	1.3	0.9	3.2	2.3	0.9	0.8
3	2.5	1.8	6.2	4.4	1.7	1.6
4	3.2	2.3	7.9	5.6	2.1	2.1
5	3.6	2.6	9.0	6.3	2.4	2.3
6	4.0	2.9	10.1	7.1	2.7	2.6
IS severity	Mild: 42%; Moderate: 26%; Severe: 10%; Fatal: 22%					

*AF* atrial fibrillation, *IS* ischemic stroke, *NOAC* non-vitamin K oral anticoagulants

*NOAC was used as treatment in base case, and warfarin was considered in sensitivity analysis; ^†^class-effect for NOAC was assumed by taking the average efficacy of apixaban, dabigatran (low and high dose), rivaroxaban, edoxaban (low and high dose) [14]. IS risk was adjusted by a factor of 1.46 (95% CI 0.8–2.16) per decade [13]; ^‡^Patients with CHADS_2_ ≤ 2were not included in our analysis

### Bleeding events: NOAC cessation and QoL impact

NOAC treatment carries a bleeding risk, which was weighted in the model by age as per a previous cost-effectiveness publication [17]. Bleed severity (see Additional file 1: Sect. 5.2 and Table S13) was independent of the particular anticoagulation treatment given [18]. Non-fatal extracranial hemorrhage (ECH) was assumed to cause cessation of NOAC therapy in the patients who were receiving NOAC at the time of the bleed. For 75% of patients with ECH, NOAC discontinuation was for 6 weeks only; for the remaining 25% the discontinuation was permanent. Patients who experienced the ECH while receiving aspirin or no treatment were barred from future NOAC use in 25% of cases [19, 20]. Similarly, intracranial hemorrhages (ICH) that were not HS, triggered temporary (6 week) NOAC discontinuation in 44% of patients on NOAC and permanent discontinuation in 56%. Patients on aspirin or no treatment at the time of an ICH were precluded from future NOAC use in 56% of cases [19, 20]. NOAC was not discontinued in the event of a clinically relevant non-major (CRNM) bleed. HS led to permanent NOAC discontinuation in 100% of patients on NOAC, and exclusion from any future NOAC use in all categories of patient, regardless of their AF status, AF diagnosis or therapy received. Additionally, there was an annual NOAC discontinuation rate of 14.8% for reasons unrelated to bleeding events [17].

A HS, like an IS, was assumed to have a permanent impact on a patient’s healthcare costs, QoL and mortality risk (Additional file 1: Sect. 7 and Table S17). The effects of a non-fatal ECH, other ICH (i.e. non-hemorrhagic stroke) and CRNM bleed were temporary, applied only during the cycle when the bleed occurred.

### Mortality for causes other than cerebrovascular events

All patients had an age-related all-cause mortality risk in the background, based on US life tables but adjusted to remove risk attributable to cerebrovascular events (which the model already covers) [21, 22]. Following a non-fatal IS or HS, this mortality risk was assumed to increase depending on stroke severity and the anticoagulation treatment currently received (Additional file 1: Sect. 6).

### Health-related QoL

Patient’s mean QoL, expressed as a utility value between 0 and 1, was sourced from EQ-5D QoL questionnaires collected at baseline in the REVEAL AF study. The OXVASC study and Gage 1996 were then used to estimate the expected QoL decrement (or disutility value) associated with strokes and their aftermath. Previously published health economic analyses were used to obtain estimates of disutility or utility reduction associated with various bleeds [16, 17, 23, 24]. The mean utilities used, and further details on their calculation are provided in Table 3.

**Table 3:** Cost and utilities of interventions, events and health states

Event, intervention or health state	Mean cost ($)	SE ($)	Mean utility	SE	Source
*Stroke and bleed events†*					
Mild IS	21,567	3301	0.76	0.05	[24–26]
Moderate IS	25,124	3846	0.39	0.02	
Severe IS	32,279	4941	0.11	0.01	
Fatal IS	32,279	4941	0.00	–	
Mild HS	23,622	3616	0.76	0.05	
Moderate HS	33,408	5114	0.39	0.02	
Severe HS	43,195	6611	0.11	0.01	
Fatal HS	43,195	6611	0.00	–	
Disutility for all recurrent (secondary) stroke events (acute period)	–		− 0.150	0.040	[23, 24]
*Other events*					
Cost and disutility of other ICH	25,149	3849	− 0.181	0.014	[16, 17, 26, 27]
Cost and disutility of CRNM	1163	178	− 0.181	0.014	
Cost and disutility of GI bleed	9136	1398	− 0.181	0.014	
Cost and disutility of other ECH	13,813	2114	− 0.181	0.014	
*Health states before any event*					
Starting utility and No AF	–		0.81	0.008	[27]
Disutility for presence of AF	–		− 0.014	0.019	[24]
*Post-stroke health states (per cycle)*					
Post mild stroke (IS or HS)	3239	496	0.76	0.012	[24–26]
Post moderate stroke (IS or HS)	8845	1354	0.45	0.035	
Post severe stroke (IS or HS)	19,212	2941	0.34	0.065	
Disutility for recurrent (secondary) stroke (post-acute period)	–		− 0.068	0.024	[23, 24]
*One-time intervention costs*					
ICM acquisition and insertion	7042	1078	–	–	[28]
ICM removal	738	113	–	–	[28]
24-h Holter	102	16	–	–	[28]
ECG device	27.5	4	–	–	[28]
*Monitoring and follow-up (per cycle)*					
ICM	119		–	–	[28, 29]
SoC monitoring	35		–	–	[28, 30]
*Drug costs (per cycle)*					
Aspirin	54		–	–	[30]
Warfarin	18		–	–	[31]
Warfarin INR monitoring	25		–	–	[28]
NOAC^*^	118		–	–	[30]

*AF* atrial fibrillation, *ECH* extracranial haemorrhage, *GI* gastrointestinal, *HS* hemorrhagic stroke, *ICH* intracranial haemorrhage, *ICM* insertable cardiac monitor, *INR* international normalised ratio, *IS* ischemic stroke, *NOAC* non-vitamin K oral anticoagulants, *SE* standard error, *SoC* standard of care

*NOAC drug cost was assumed to be the average of dabigatran, rivaroxaban, apixaban, and edoxaban; ^†^confidence intervals associated with costs are assumed to be + / − 30%, utility values were updated for the heart failure subgroup, as detailed in the Additional file 1: Table S17

### Resource use and costs

#### Device and monitoring costs

Costs of purchasing, inserting and later removing the ICM following battery expiry (after 3 years) were informed by U.S. Medicare 2020 national average payer paid amounts [28]. There were also per-cycle costs for in-person and remote clinician visits. The number of visits was informed by informed by the LINQ Registry study [29] and the costs were informed by U.S. Medicare 2020 national average payer paid amounts [28]. Patients who did not have AF detected continued to receive SoC after ICM removal. Unplanned ICM removal before battery expiry due to a range of clinical, technical, or personal factors occurred at a rate of 2.9% per year based on REVEAL AF (Medtronic data on file).

Patients receiving SoC in the model received 0.17 24-h Holter monitors and 3.40 ECGs annually on average. This was informed by real-world claims data for a cohort with similar characteristics [30]. The unit costs of this monitoring were estimated from U.S. Medicare 2020 national average payer paid amounts [28].

The base case used public payer costs taken from Medicare [28], and a scenario analysis, in which device and monitoring costs were increased by 25%, was performed to model a hypothetical cohort of 100% private payer patients.

#### Treatment and event-related costs

The costs of oral anticoagulation were estimated using data from the national average payer paid amounts (Additional file 1: Table S19).

Strokes and bleeds had high one-time costs associated with managing the event, and where they caused permanent health consequences, additional per-cycle costs were applied in a set of “post-stroke” health states. Health state and event costs were derived from published literature [26] and adjusted where necessary to 2020 levels using the “Medical Care component of the consumer price index [32] (Table 3).

### Model output and sensitivity analysis

The incremental cost-effectiveness ratio (ICER) for ICM versus SoC was calculated using total QALYs and healthcare costs over the cohort lifetime. Results were also generated for a hypothetical cohort of 1000 patients. Deterministic sensitivity analyses were undertaken to assess the impact of assumptions about the method and accuracy of AF detection, OAC treatment uptake and discontinuation, the type of OAC therapy that is given following AF detection (warfarin versus NOACs) and the costs of detection and medication. Probabilistic sensitivity analysis, varying all model parameters simultaneously within appropriate distributions, was performed storing the results of 1000 samples. Cost-effectiveness was tested at the $50,000 and $150,000 thresholds, in keeping with US guidelines [33].

### Subgroup analysis

**Fig. 3:**
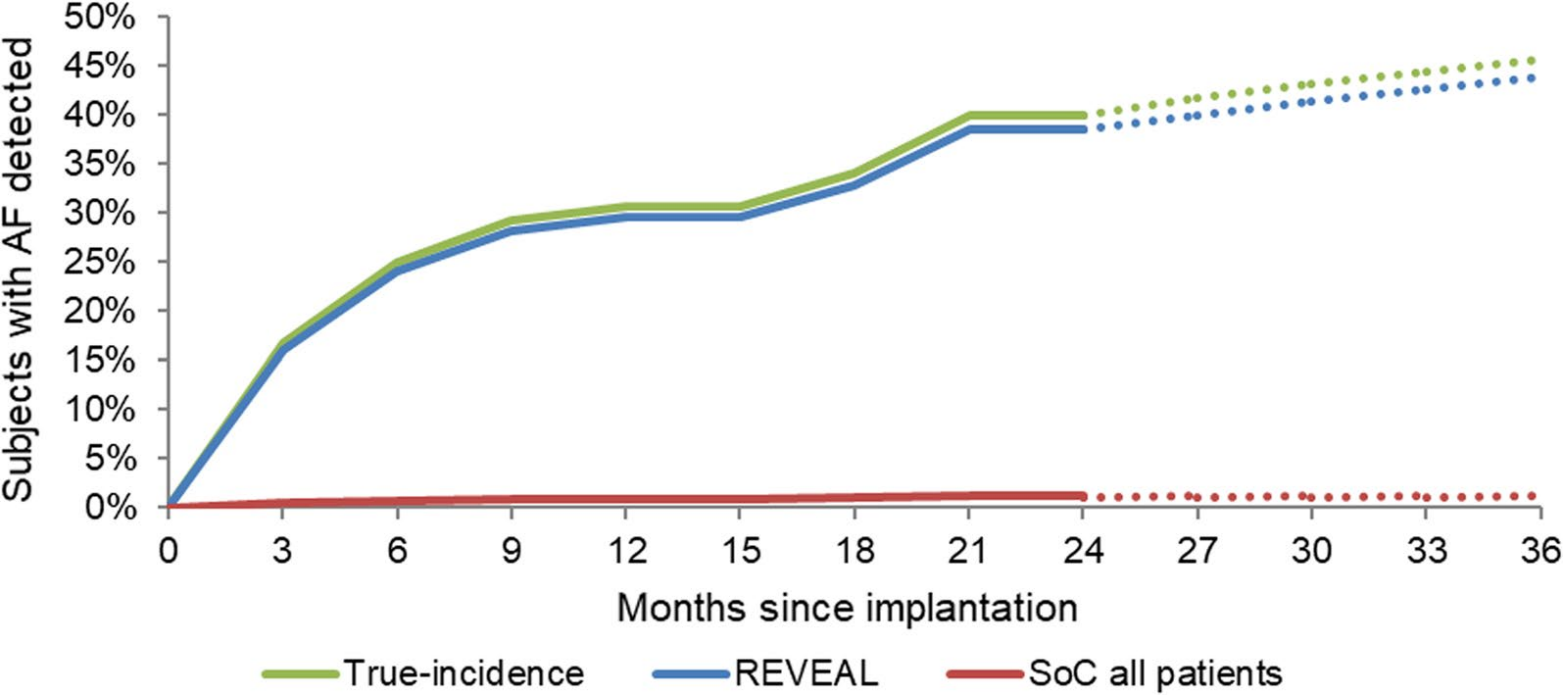
AF detection rates in ICM and SoC—heart failure subgroup. *Notes*: Both ICM and SoC detection curves are modelled based on analysis of the REVEAL AF trial dataset (5). The AF burden is extrapolated from the 24 months of study data (after which it becomes underpowered) and the end of the battery life at 36 months, using a linear extrapolation from month 3 to month 30 (3.9% per 3-month cycle). Abbreviations: AF: atrial fibrillation; ICM: insertable cardiac monitor; SoC, standard of care

Subgroup analyses by CHADS2 score and by history of heart failure (Fig. 3) were performed, using AF detection and patient demographic data from REVEAL AF. In addition to using the REVEAL subgroup data, the modelling of patients with a history of heart failure required an updated set of bleed risks associated with NOACs, derived from relevant subgroup analyses from published studies (Additional file 1: Table S11).

## Results

### Base case

The base-case analysis (Table 4) found that across patients’ lifetimes, the ICM strategy provided an additional 0.16 QALYs compared to SoC. The incremental cost was $5786, giving an ICER of $35,528 per QALY gained. There were 34 fewer ischemic strokes per 1000 patients in the ICM group compared to SoC, and an overall life-year gain of 0.17. These results suggest that ICM is a cost-effective monitoring strategy compared to SoC in high-risk AF patients. The model estimates a number needed to treat (NNT) of 29 per stroke avoided.

**Table 4:** Base-case results

	SoC	ICM	Difference
*Deterministic analysis*			
Total costs	$25,330	$31,116	$5786
Total IS per 1000 patients	277	243	34
QALYs	7.59	7.75	0.16
Life years	9.72	9.98	0.17
ICER ($/QALY)			$35,528
ICER ($/LY)			$33,641
*Probabilistic sensitivity analysis (average 95% CrI)*			
Total costs	$26,035 ($20,452 to $33,532)	$31,836 ($26,069 to $38,285)	$5801 ($3,522 to $8,264)
Total IS per 1000 patients	282 (180 to 405)	248 (156 to 356)	− 34 (− 19 to − 55)
QALYs	7.48 (5.95 to 8.48)	7.64 (6.10 to 8.61)	0.16 (0.07 to 0.29)
Life Years	9.69 (9.13 to 10.14)	9.87 (9.38 to 10.25)	0.18 (0.09 to 0.30)
ICER ($/QALY)			$35,774

*CrI* credible interval, *ICM* insertable cardiac monitor, *IS* ischemic stroke, *LYs* life years, *QALYs* quality-adjusted life years, *SoC* standard of care

ICM had higher monitoring costs compared to SoC at initiation and during the 3 years of regular follow-up. Furthermore, bleed-related costs for the ICM strategy ($6803) were slightly higher than for SoC ($6302) because more patients on ICM had AF detected and went on to receive anticoagulation. However, costs related to IS were reduced in the ICM group compared to SoC ($7204 vs. $8225 respectively), since the improved AF detection in the ICM group ultimately led to the prevention of more stroke events (and their related costs). Other health state costs were also lower for ICM than for SoC ($8246 vs. $8609), due to lower post-stroke health costs. Overall, purchasing an ICM reduced costs in other areas.

**Fig. 4:**
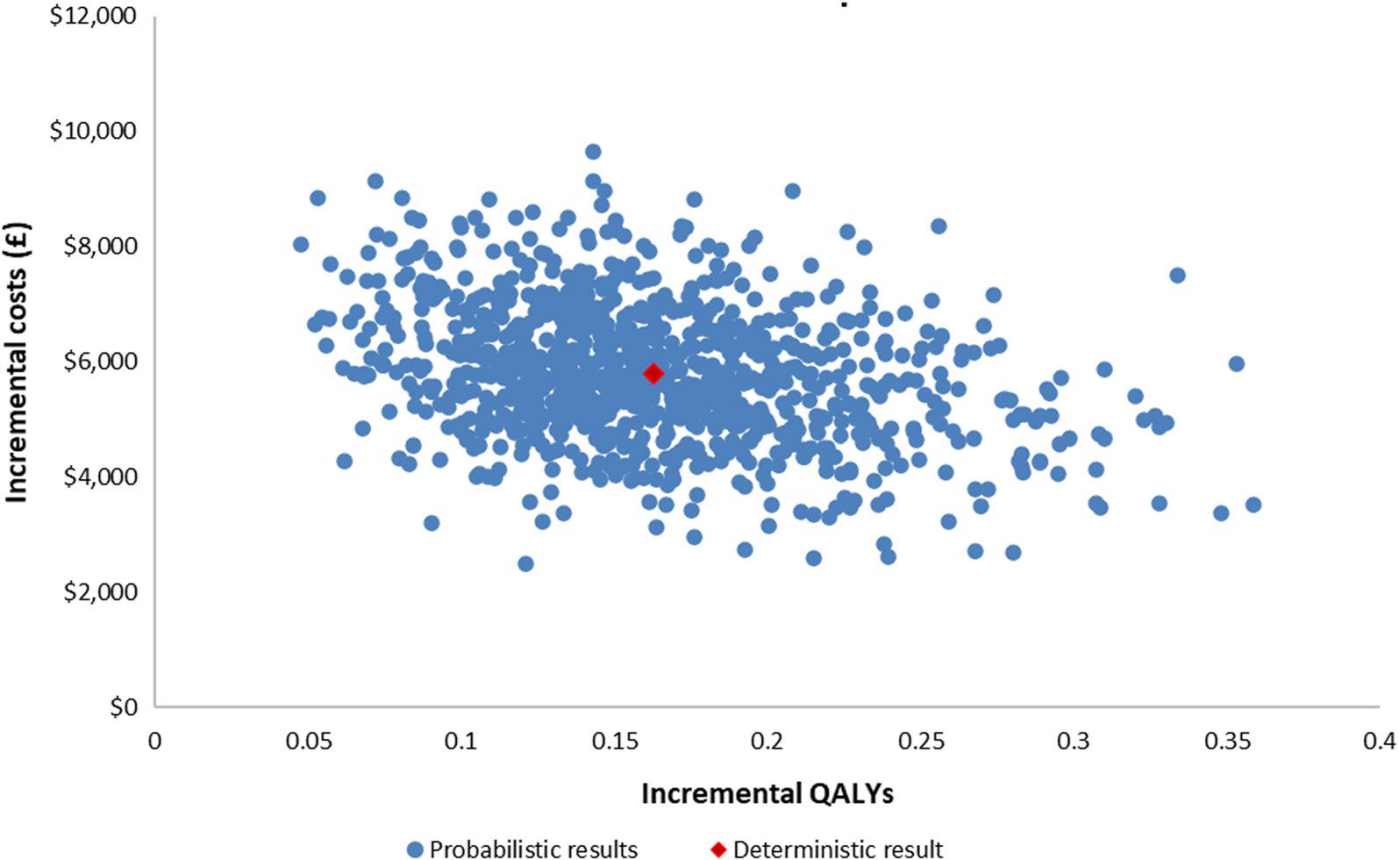
Scatter plot

**Fig. 5:**
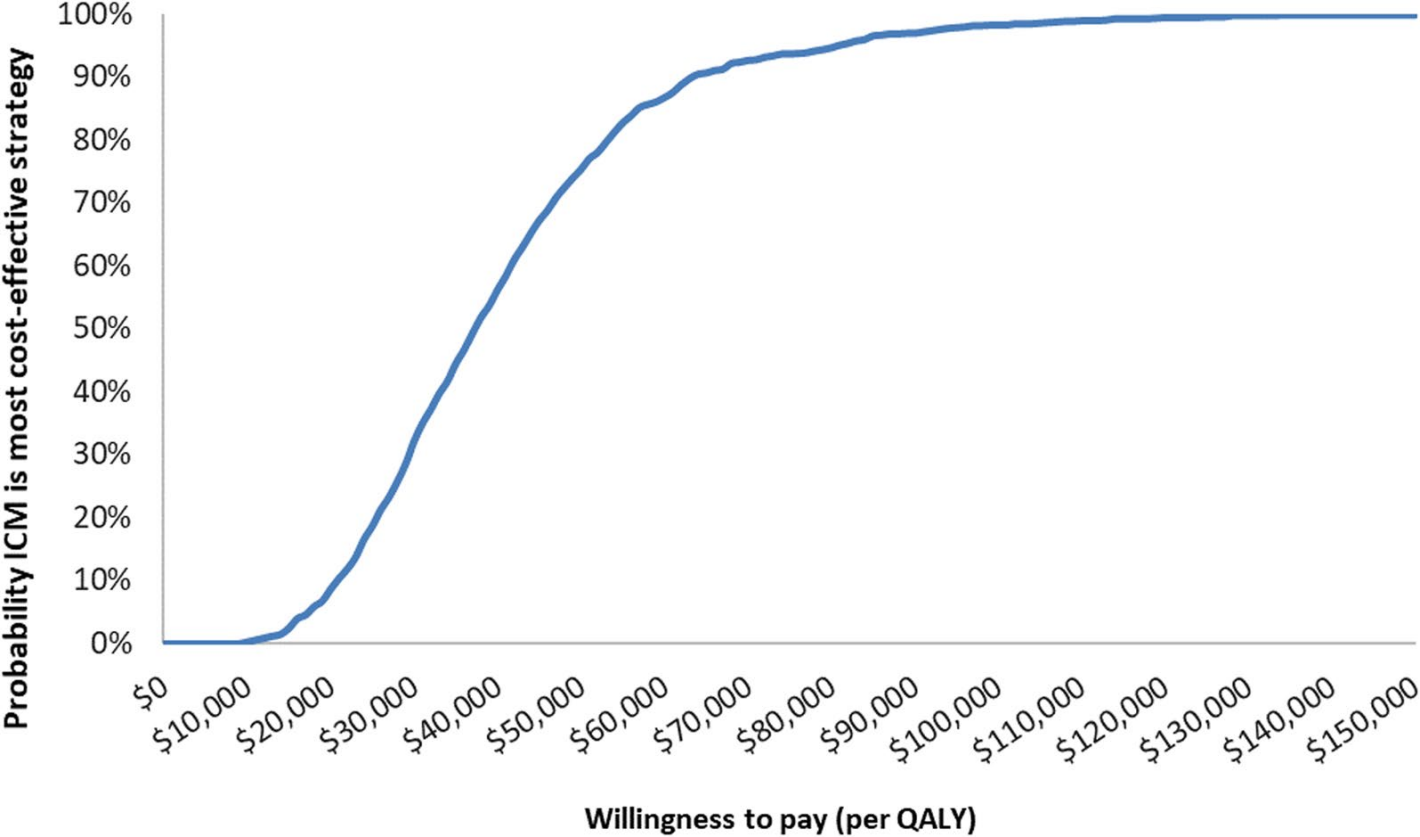
Cost-effectiveness acceptability curve

Probabilistic sensitivity analysis found ICM had a 75% probability of being highly cost-effective at a willingness-to-pay (WTP) threshold of $50,000 per QALY, and a 100% probability of being cost-effective at a WTP threshold of $150,000 per QALY (Figs. 4, 5).

### Subgroup analyses

The base case assumed a distribution of patient CHADS_2_ scores matching baseline characteristics in the REVEAL AF study. The analysis was repeated, restricting to each CHADS_2_ subgroup (see Table 5). The ICER in lower-risk patients (CHADS_2_ score = 2) was higher than the base case ($55,059 per QALY). The ICER in the group with CHADS_2_ score = 3 was similar to the base case (the base-case population had a mean CHADS_2_ score of 2.94). ICERs for patients with higher-risk CHADS_2_ scores (4, 5 or 6) were slightly higher than the base case, with smaller incremental costs and smaller QALY gains.

**Table 5:** Subgroup analysis by CHADS2 score and scenario analyses

Scenario description	ICER per QALY		
	Incremental cost	Incremental QALYs	ICM versus SoC
*Base case*	$5786	0.16	$35,528
Sub-group analyses^*^			
CHADS_2_ score 2	$6409	0.12	$55,059
CHADS_2_ score 3	$5867	0.16	$37,118
CHADS_2_ score 4, 5, and 6	$5792	0.13	$43,803
*Scenario analyses*			
Choice of NOAC = warfarin	$5950	0.11	$56,916
Cost of aspirin = $0	$5917	0.16	$36,328
Treatment discontinuation for reasons other than bleeding = 0%	$4933	0.31	$15,843
ICM battery life 4.5 years	$5777	0.17	$33,229
ICM battery life 4.5 years, assume linear extrapolation	$5786	0.20	$27,986
ICM battery life 4.5 years, assume no AF after 30-month trial data	$5806	0.16	$37,594
Monitoring costs for SoC: assume pulse check and HR of ICM versus SoC is 1/24th of the Holter monitoring (scenario proposed by clinical experts)	$6954	0.17	$40,992
Assume SoC consists of one 24-Holter in the first cycle	$6773	0.17	$31,116
AF episode duration lasting for ≥ 5.5 h^†^	$6713	0.08	$86,013
NOAC uptake after AF diagnosis = 66.35%	$6301	0.12	$54,368
Time horizon = 5 years	$6433	0.02	$261,147
Time horizon = 10 years	$5797	0.08	$75,047
Time horizon = 25 years	$5776	0.16	$35,771
*Heart failure subgroup base case*	$6236	0.19	$33,273
*Scenarios*			
Choice of NOAC = warfarin	$6384	0.13	$50,659
ICM battery life 4.5 years	$6193	0.20	$30,777
ICM battery life 4.5 years, assume linear extrapolation	$5837	0.28	$20,967
ICM battery life 4.5 years, assume no AF after 2-year trial data	$6508	0.19	$34,332

*AF* atrial fibrillation, *HR* hazard ratio, *ICER* incremental cost-effectiveness ratio, *ICM* insertable cardiac monitor, *NOAC* non-vitamin K oral anticoagulants, *QALY* quality-adjusted life-year, *SoC* standard of care

*Each CHADS_2_ subgroup will differ on ischemic stroke risk, diagnostic accuracy of monitoring strategies, as well as the corresponding age and gender mix of the group in the REVEAL AF trial; ^†^analyses using the alternative definition of AF episode were carried out using the REVEAL AF clinical dataset (Data on file, Medtronic 2018)

A subgroup analysis was performed in the 81 REVEAL AF study patients who had a history of heart failure. The ICER for this subgroup was $33,273, which is lower than in the base case, driven by a higher rate of AF detection (43.8% at 3 years) and higher average CHADS_2_ score.

### Sensitivity and scenario analyses

**Fig. 6:**
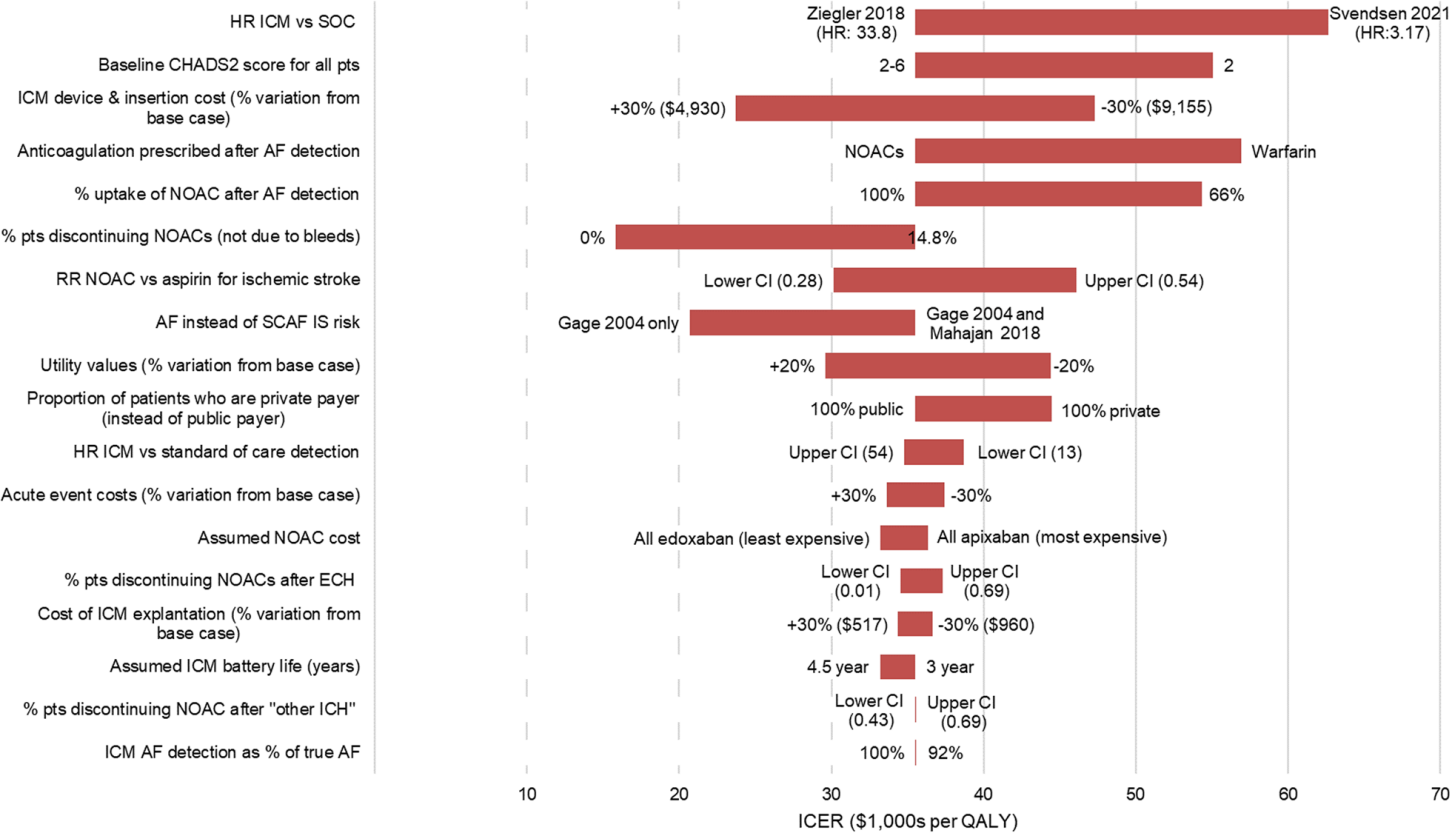
Tornado diagram. *Notes*: Bars reflect the extent of ICER impact of the low and high limits for parameter intervals considered; the value or source for each alternative parameter value is shown at the ends of each bar

Table 5 presents the main parameters and scenarios tested, alongside their impact on model results. The tornado diagram (Fig. 6) visually summarizes the results of key single-parameter sensitivity analyses and scenario analyses.

The primary efficacy parameter for this model was the rate of AF detection with ICM, in relation to SoC. In the base case, ICM was modelled to detect AF at a rate 33.9 times that of SoC. The ICER increased slightly (to $38,723 per QALY) when this ratio was set to its lower confidence limit of 13. The recent LOOP study by Svendsen et al. estimated a ratio of 3.17 between ICM and SoC for AF detection efficacy [34]. When this substantially lower ratio was substituted into the model, the ICER increased to $62,662 per QALY.

Using an alternative definition of AF (i.e. requiring a 5.5-h episode captured by ICM to trigger AF diagnosis) produced a higher ICER ($86,013).

In the base case, IS risk was assumed to increase 2.4-fold following a subclinical, device-detected AF diagnosis (based on Mahajan et al. [15]). When this relative risk figure was instead taken from a study (Gage et al. [1, 12]) that used more acute clinical, ECG-detected AF (RR = 4.8), the ICER fell to $20,717per QALY.

Costs of the ICM device and its insertion procedure impacted the ICER substantially, with a 30% cost increase driving the ICER up to $47,320 per QALY, and a 30% cost decrease reducing it to $23,735 per QALY. In the base case it was assumed that all patients received Medicare funded healthcare. Assuming 100% of patients receive private payer healthcare, resulted in an ICER of $44,479. Also linked to ICM costs but affecting a wider range of outcomes, we tested scenarios where the ICM battery lasted 50% longer (4.5 years vs. 3 years in the base case). Under these longer battery-life scenarios, the ICER decreased to $33,229, with 37 fewer strokes per 1000 patients in the ICM arm compared to SoC. This “4.5 years battery life” scenario was further explored using alternative methods of extrapolating AF detection data beyond the 30 months’ follow-up from the REVEAL AF trial. Linear extrapolation yielded an ICER of $27,986, whereas assuming no further AF detection beyond 30 months yielded an ICER of $37,594.

Uptake, efficacy and safety of NOAC therapy after AF detection have substantial effects on the ICER, as does the discontinuation rate for NOAC. Where NOAC uptake was assumed to be lower (66.35% vs. 100% in the base case), the ICER rose to $54,368 per QALY. If NOAC efficacy in reducing IS risk is assumed to be better (RR = 0.28 NOAC vs. aspirin, compared to 0.39 in the base case) the ICER improves to $30,128 per QALY. If the less-expensive warfarin is administered instead of NOACs, the ICER rises to $56,916 due to the increased hemorrhage risk, which causes QALY losses and net cost gains from treating bleeding events. Varying the assumed cost of NOACs, whilst holding their efficacy constant, resulted in an ICER of between $33,239 and $36,360. The base case allowed a range of reasons for NOAC discontinuation; however, the ICER improved to $15,843 per QALY if discontinuation was only allowed in the event of bleeds—this alternative discontinuation rule meant more patients remained on NOAC therapy and thus experienced reduced IS risk.

Several of these key scenarios were repeated for the higher-risk heart failure subgroup (Table 5). The direction of change was always consistent with that observed in the all-patient population. For example, when ICM battery life was increased to 4.5 years in the heart failure subgroup, the ICER fell to $30,777. When this battery-life scenario was subdivided by AF data extrapolation method, the linear extrapolation method yielded an ICER of $20,967, whereas no extrapolation (i.e. no AF beyond 30 months) yielded an ICER of $35,332.

## Discussion

This study modelled the indirect relationship between ICM use (a diagnostic strategy) and stroke reduction (event avoidance) via appropriate oral anticoagulation (improved treatment). Consistent with previous models in this area, the cost-effectiveness of ICM is driven by its diagnostic advantage over SoC, and the extent to which this influences subsequent health events and their costs. While implanted, ICM has been estimated to detect AF at a rate over 33 times higher than SoC on average. This allows a far greater proportion of patients to be prescribed NOACs, which in the model led to fewer strokes, improved life expectancy and higher quality of life. There were slightly greater costs related to bleeds in the ICM group, but these were offset by long-term savings associated with reduced IS. In the base case analysis, ICM was a cost-effective way to monitor for AF in a high-risk population within the US healthcare setting, with a mean ICER below the commonly-accepted lower threshold of $50,000 per QALY gained [35].

This is the first model of its kind developed for the US. It was adapted from a previous model developed in the UK [8] but with several key modifications. The UK model calculated IS risk based on a longitudinal study of the association of clinical, ECG-detected AF with IS [1]; thus each AF event substantially increased IS risk (RR for AF = 4.8). By contrast, this US model referenced a more relevant study into the association of subclinical, device-detected AF and IS [15]. Using this source, each AF event is associated with a lower, 2.4-fold increase in IS risk. An additional difference is that the SoC monitoring frequency in this model was informed by real-world data collected in a US setting [30]. Compared to a previous US-based model involving ICM in patients with cryptogenic stroke, the ICER from this analysis was slightly higher [36].

The main sensitivities explored for the model related to ICM diagnostic efficacy, longevity and cost, IS risk associated with AF, NOAC treatment and its various dimensions, as well as subgroup analyses.

Diagnostic accuracy of AF diagnosis through ICM versus SoC had only a modest effect on cost-effectiveness when varied based on confidence intervals from the publication source used. Assuming no change in other parameters, we determined that ICM only has to detect AF at about 4.5 times the rate of SoC to be considered cost-effective at the most stringent threshold of $50,000 USD per QALY considered ‘highly cost-effective.’ The recent LOOP study in the Danish healthcare setting [34] found a lower relative difference in AF detection rates between ICM and SoC. When this rate was applied in the model, it gave an ICER of $62,662. However, the LOOP study estimated a uniquely high rate of AF detection in its SoC arm which may not reflect the detection that could be expected in clinical practice. The particularly high levels of cardiac monitoring in the SoC arm may have been impacted by the unblinded nature of the study, and additionally may not be generalizable outside of the Danish setting. Moreover, the study conclusions regarding the general effectiveness of ICM in preventing strokes remain uncertain due to the ongoing unresolved equipoise around the stroke reduction of OAC therapy administered after device-detected AF, compared with ECG-detected AF. This issue may be resolved once the stroke outcome-based RCTs of ICM, ARTESIA and NOAH [37, 38], are completed.

Newer ICMs carry an extended battery life of 4.5 years. To reflect this greater battery longevity, our model extrapolated the extent of additional AF detection after the study duration of REVEAL AF. Changing assumed rates of AF detection at timepoints beyond the 30 months of observation in the REVEAL AF trial had a moderate impact on the ICER, although all tested assumptions produced an ICER which remained below the $50,000 cost-effectiveness threshold. Additionally, since ICMs represent a potential lifetime strategy for monitoring AF, the assumptions made in this model around monitoring durations and explantation could be programmed in a more sophisticated fashion if additional data become available. If new trials are conducted with longer time horizons of monitoring and managing AF, this would likely yield improved cost-effectiveness estimates for ICM use.

In our model, the diagnostic benefit of ICM is translated into cost and QALY benefits via the use of NOACs, which reduce IS risk. Real-world cost-effectiveness will be determined by how closely US clinical anticoagulation practices (and their results) reflect the model. For example, we assumed that all patients receive NOACs immediately upon AF detection, and that 14.8% of them discontinue treatment annually for reasons other than bleeding. It is possible that in practice, rates of anticoagulation uptake and patient compliance are lower, thus leading to poorer protection against strokes than we have assumed. Real-world estimates for NOAC uptake following AF diagnosis vary substantially, from 76 to 100% [39–41]. The model remained relatively robust to less favourable assumptions; annual discontinuation for reasons other than bleeds would need to exceed 26% for the ICER to exceed the $50,000 WTP threshold.

The assumed efficacy of anticoagulation therapy also impacted cost-effectiveness, but the ICER withstood modest changes in the hazard ratio for NOAC efficacy. Furthermore, the relative clinical benefit of NOACs in stroke prevention is well established [14]. When the higher-risk warfarin was substituted as anticoagulation in scenario analysis, the ICER worsened but remained below the accepted WTP threshold.

Following a major bleed, clinicians must decide whether it is worth the risk of allowing patients to continue NOAC therapy, or if it is safer to stop. The exact approach taken can vary in the US clinical setting. The model took a conservative approach where hemorrhagic strokes led to immediate and permanent discontinuation of NOACs with no exceptions. Future analyses could explore alternative approaches here, for example allowing cautious use of NOACs in limited circumstances despite the occurrence of hemorrhagic strokes (either in the patient’s past history or during the modelled NOAC treatment phase).

Cost-effectiveness of ICM would normally be expected to improve in groups with higher underlying AF risk, because there should be more potential AF episodes to detect, which would then lead to more AF diagnoses and initiations of NOAC treatment, resulting in more IS events avoided. This pattern was partly consistent when we restricted our model by CHADS_2_ subgroups (values 2, 3 and 4–6). ICM was least cost effective in the lowest-risk group (CHADS_2_ = 2), and similar to base-case for CHADS_2_ = 3. However, the ICER was not improved for patients in the highest-risk CHADS_2_ groups (scores 4–6), indeed it was slightly higher than base-case. This counterintuitive result is explained by the REVEAL AF trial study data, wherein fewer patients in CHADS2 groups 4–6 received a confirmed AF diagnosis compared to the whole population. A larger real-world study would be expected to clarify this uncertainty, which may in part be due to the lower sample sizes collected in these subgroups.

In the heart failure subgroup, ICMs were found to be more cost-effective. This was mainly due to the higher rate of AF detection within the subgroup along with their higher average CHADS_2_ score [37, 38].

In a zero-cost scenario where SoC was assumed to comprise a pulse check and identified 1/24th of the AF detected by SoC in the base case, the ICER for ICM rose to $40,992 per QALY. In a scenario where SoC was assumed to comprise only a one-off 24-h Holter monitor, the ICER for ICM rose to $40,323. In practice, SoC can vary substantially, with patients receiving different monitor lengths with varying regularity; the cost-effectiveness of ICMs is robust to variations in clinical practice [39–41].

### Limitations

First, the benefit of anticoagulation in reducing stroke risk among patients with clinical AF is well established. This paper modelled the benefits of anticoagulation in patients with device-detected AF, which is a less well-studied risk factor for IS. Both patients’ stroke risk (i.e., their CHADS_2_ score), and the efficacy of NOACs in reducing stroke risk, were varied in sensitivity analysis. Cost-effectiveness estimates were robust to changes in these parameters. Several ongoing studies will provide additional estimates of the absolute stroke reduction benefit of screening patients for subclinical AF using device detection, reducing uncertainty in this area [37, 38].

Second, this study modelled whether patients had AF diagnosed by an ICM, but not the extent of their AF burden. Increasing the threshold for AF diagnosis from 6 min to 5.5 h had limited impact on cost effectiveness, providing evidence that results were robust. The link between AF burden and IS risk remains uncertain. It is worth noting that elevated stroke risks have been observed in patients with short AF episodes of ≥ 6 min (irrespective of clinical diagnosis) [42] as well as in patients with ≥ 5.5 h AF episodes (detected via a cardiovascular implanted electronic device) [43]. The REVEAL study patient population had a wide distribution of AF episode lengths triggering diagnosis. Of the 128 patients with AF lasting 6 or more minutes, 113 (88.3%) had 30 or more minutes of AF, 97 (75.8%) had 1 or more hours of AF, and 53 (41.4%) had 6 or more hours of AF in a day at some point. Thirteen patients (10.2%) had at least one episode lasting 24 h or longer. Modelling a more sophisticated relationship between this extended range of AF burdens and stroke risk could give a clearer understanding of which patients may benefit most from anticoagulation. The real-world outcomes associated with more systematic screening for AF have been reinforced by new research [44], which could also form a useful validation reference for any such new models.

This study did not estimate the population health impact of increasing the use of ICMs among patients at high risk of atrial fibrillation. A future study focusing specifically on population health impact could help to further inform payer decision making regarding ICMs.

### Further research

An updated version of CHADS_2_, called CHA_2_DS_2_-VASc, has recently been introduced [17]. This score was not captured in the REVEAL AF trial, but future models could potentially make use of a mapped version of this new risk score.

Extending the model with real-world observations from longer-term monitoring or building a model with greater flexibility to simulate alternative assumptions around AF burden and detection over time, could help identify which factors are the most important determinants of cost-effectiveness in ICM monitoring.

## Conclusions

This analysis found ICM to be a cost-effective method for detecting AF in high-risk populations in the US healthcare setting, because of the benefit it can provide through timely diagnosis and anticoagulation to prevent strokes. ICM use is particularly more cost effective in patients who also have a history of heart failure. The model was developed for the US, but findings align with models developed for other countries (for example the UK [8] and for the related population of cryptogenic stroke patients [45]).

Long-term monitoring for AF and associated stroke prevention form part of an evolving field. There are regular updates to risk stratification methods, available anticoagulation medicines, and AF detection technology. The sensitivity analyses conducted suggest that most therapeutic and diagnostic advancements will further increase the cost-effectiveness of ICMs.

## Supplementary Information

**Additional file 1.** Supplementary information.

## Data Availability

Data are available upon request by contacting the corresponding author: lsawyer@symmetron.net.
