# Supplementary material for: Cost-effectiveness of an insertable cardiac monitor in a high-risk population in the US
Source: BMC Cardiovasc Disord. 2023 Jan 25;23:45. doi: 10.1186/s12872-023-03073-6 (PMC9875401; doi:10.1186/s12872-023-03073-6)
Supplement: Supplementary file 1 — Additional file 1. Supplementary information. [file 12872_2023_3073_MOESM1_ESM.docx]

Supplementary Data

# Hypothetical cohort characteristics

The model considered a hypothetical cohort of patients with baseline characteristics as observed in the REVEAL AF clinical trial (Table 1). The average CHADS_2_ score of the population entering the economic model is 2.94. The baseline characteristics for the heart failure subgroup analysis were informed by the relevant subgroup of patients within the REVEAL AF clinical trial also (Table 1). Inputs for this subgroup were the same as for the all-patient population, unless stated otherwise.

Table 1. REVEAL AF clinical trial cohort characteristics – all patients

| **CHADS_2_ score** | **CHADS_2_ 2** | **CHADS_2_ 3** | **CHADS_2_ 4** | **CHADS_2_ 5** | **CHADS_2_ 6** | **All patients** | **Heart-failure subgroup** |
| --- | --- | --- | --- | --- | --- | --- | --- |
| **N** | 158 | 130 | 75 | 26 | 4 | 393 | 81 |
| **Mean age** | 69.4 | 72.7 | 71.7 | 77.6 | 71.6 | 71.3 | 65.8 |
| **% male** | 56.3% | 50.8% | 49.3% | 42.3% | 50.0% | 52.0% | 66.7% |
| **Antiplatelet usage at baseline (%)** | | | | | | |  |
| Yes | 72.2% | 67.7% | 78.7% | 84.6% | 75.0% | 72.6% | 70.4% |
| **History of cerebrovascular accident (stroke) at baseline (%)** | | | | | | |  |
| Yes | 1.9% | 16.2% | 50. 7% | 57.7% | 75.0% | 20.3% | 12.4% |

Source for values in table: REVEAL AF, data on file

# Extrapolation of incidence and detection

The REVEAL AF study was 30 months long, meaning that the risk of patients experiencing AF episodes needed to be extrapolated after 30 months. A number of options were compared, and logarithmic extrapolation, excluding the first cycle was the best fit for the data. This was implemented such that the curve passed through the final observed data point. The subgroup of patients in REVEAL AF with a history of heart failure was only powered for 24 months, and AF detection was extrapolated from this point onwards.

# Subgroup incidence and detection

The AF episode duration threshold at which to initiate OAC therapy was discussed by clinical experts (co-authors KW, ME, MR); it was agreed that the threshold was dependent on other risk factors included in the CHADS_2_ score (e.g. a lower threshold might be used for patients with a history of stroke or high CHADS_2_ score, whereas a higher threshold might be appropriate for a patient with a low CHADS_2_ score). From the discussion, it was clear that this is an area of high uncertainty and that clinical opinion is likely to vary. Furthermore, 6 minutes was the threshold used in the REVEAL AF study ([1](#_ENREF_1)), while 5.5 hours has been previously used in the TRENDS data ([2](#_ENREF_2)).

Given that the model cohort enters the model with an average CHADS_2_ score of 2.94, base-case AF episodes were defined as lasting for ≥6 minutes, whilst a scenario analysis explored the impact of AF episodes lasting for ≥5.5 hours.

Table 2. Atrial fibrillation detection (%) observed in REVEAL AF trial by CHADS_2_ subgroup, ≥6 min AF episodes

| **CHADS_2_ score** | **All patients** | **Heart failure subgroup** | **CHADS_2_ 2** | **CHADS_2_ 3** | **CHADS_2_ 4, 5, 6** |
| --- | --- | --- | --- | --- | --- |
| **Timepoints (months)** | | | | | |
| Month 0 | 0.0 | 0.0 | 0.0 | 0.0 | 0.0 |
| Month 3 | 13.7 | 16.1 | 12.5 | 16.2 | 12.5 |
| Month 6 | 20.4 | 24.0 | 18.7 | 22.9 | 19.5 |
| Month 9 | 23.9 | 28.1 | 20.8 | 27.0 | 24.6 |
| Month 12 | 27.0 | 29.5 | 23.9 | 29.8 | 29.2 |
| Month 15 | 27.6 | 29.5 | 23.9 | 30.8 | 29.5 |
| Month 18 | 29.2 | 32.7 | 24.6 | 32.6 | 31.8 |
| Month 21 | 31.0 | 38.4 | 25.5 | 35.1 | 34.2 |
| Month 24 | 33.4 | 38.4 | 28.3 | 39.5 | 34.2 |
| Month 27 | 38.1 | 40.0* | 34.1 | 44.8 | 36.6 |
| Month 30 | 39.8 | 41.4* | 34.1 | 49.4 | 36.6 |
| Month 33* | 40.8 | 42.6 | 34.9 | 50.6 | 37.6 |
| Month 36* | 41.7 | 43.8 | 35.6 | 51.7 | 38.5 |
| Month 39* | 42.6 | 44.8 | 36.3 | 52.7 | 39.4 |
| Month 42* | 43.3 | 45.8 | 36.9 | 53.7 | 40.2 |
| Month 45* | 44.1 | 46.7 | 37.5 | 54.6 | 40.9 |
| Month 48* | 44.7 | 47.6 | 38.1 | 55.4 | 41.6 |
| Month 51* | 45.4 | 48.4 | 38.6 | 56.2 | 42.2 |
| Month 54* | 46.0 | 49.1 | 39.1 | 56.9 | 42.8 |

Notes: *these values are modelled, as described in Section 2. Source for values in table: REVEAL AF, data on file; Notes: these values are modelled, as described in Section 2.

Table 3. Atrial fibrillation detection (%) observed in REVEAL AF trial by CHADS_2_ subgroup, ≥5.5 hours AF episodes

| **CHADS_2_ score** | **All patients** | **CHADS_2_ 2** | **CHADS_2_ 3** | **CHADS_2_ 4, 5, 6** |
| --- | --- | --- | --- | --- |
| **Timepoints (months)** | | | | |
| Month 0 | 0 | 0 | 0.0 | 0.0 |
| Month 3 | 7.2 | 7.7 | 7.8 | 5.8 |
| Month 6 | 8.8 | 9.1 | 9.4 | 7.8 |
| Month 9 | 9.9 | 9.7 | 10.3 | 9.8 |
| Month 12 | 11.0 | 10.4 | 11.2 | 11.9 |
| Month 15 | 11.9 | 11.1 | 11.2 | 14.0 |
| Month 18 | 12.8 | 11.1 | 13.2 | 15.1 |
| Month 21 | 13.6 | 11.1 | 13.2 | 17.7 |
| Month 24 | 15.0 | 12.2 | 16.0 | 17.7 |
| Month 27 | 18.8 | 15.7 | 17.7 | 24.3 |
| Month 30 | 19.5 | 15.7 | 20.1 | 24.3 |
| Month 33* | 20.0 | 16.0 | 20.5 | 25.1 |
| Month 36* | 20.4 | 16.3 | 20.9 | 25.8 |
| Month 39* | 20.8 | 16.5 | 21.3 | 26.4 |
| Month 42* | 21.2 | 16.8 | 21.7 | 27.0 |
| Month 45* | 21.5 | 17.0 | 22.0 | 27.6 |
| Month 48* | 21.9 | 17.2 | 22.3 | 28.1 |
| Month 51* | 22.2 | 17.3 | 22.6 | 28.6 |
| Month 54* | 20.0 | 16.0 | 20.5 | 25.1 |

Notes: *these values are modelled, as described in Section 2. Source for values in table: REVEAL AF, data on file; Notes: these values are modelled, as described in Section 2.

The hazard ratios of the ICM vs. SoC are presented in Table 4, for each of the populations considered in the economic model. Relative diagnostic yield of ICM versus SoC in the base case was sourced from a simulated comparison of AF monitoring strategies using trial data from REVEAL AF ([3](#_ENREF_3)). The authors used the ICM data from REVEAL AF to compute the AF incidence and simulate detection by one-time intermittent monitoring strategies over various recording periods following insertion of the ICM. The observed incidence of AF by continuous monitoring with ICM was compared to the simulated incidence of AF by a one-time 24-hour Holter monitor to estimate a hazard ratio of 33.9.

Note that for sub-analyses by CHADS_2_ score, since no information was available from Reiffel et al*.* ([3](#_ENREF_3)) for the CHADS_2_ subgroups in the REVEAL AF trial, values observed in the CRYSTAL-AF randomised trial comparing ICM with SoC following cryptogenic stroke were used instead ([4](#_ENREF_4)).

Table 4. Hazard ratios for ICM vs. SoC for AF detection

| **CHADS_2_ score** | **Hazard ratio (95% CI) ICM vs. SoC** | **Notes** | **Source** |
| --- | --- | --- | --- |
| All patients (base case) | 33.9 (13.2 – NE) | Estimated from a comparison of observed and simulated AF monitoring strategies using REVEAL AF data. An upper limit of 54.5 is used, which assumes that the confidence interval is symmetrical. | ([3](#_ENREF_3)) |
| CHADS**_2_** 2 | 39,000,000 (0.00 to -)* | Based on the observed data from CRYSTAL-AF, an RCT in patients with cryptogenic stroke. | ([4](#_ENREF_4), [5](#_ENREF_5)) |
| CHADS**_2_** 3 | 4.89 (1.41 to 16.9) |  | ([4](#_ENREF_4), [5](#_ENREF_5)) |
| CHADS**_2_** 4, 5, 6 | 8.49 (1.97 to 36.5) |  | ([4](#_ENREF_4), [5](#_ENREF_5)) |

*Zero cases of AF were detected with standard of care (SoC)

Abbreviations: AF, atrial fibrillation; CI, confidence interval; ICM, insertable cardiac monitor; NA, not applicable; RCT, randomised controlled trial; SoC, standard of care

Table 5. Calculated per 3-month cycle probabilities of all AF and detected AF by diagnostic strategy, ≥6 min AF episodes (%)

| **CHADS_2_ score** | **All patients** | **Heart failure subgroup** | **CHADS_2_ 2** | **CHADS_2_ 3** | **CHADS_2_ 4, 5, 6** |
| --- | --- | --- | --- | --- | --- |
| **Cycles (3-month intervals)** | | | | | |
| Cycle 1 | 13.69 | 16.09 | 12.53 | 16.22 | 12.53 |
| Cycle 2 | 7.71 | 9.38 | 7.08 | 7.92 | 7.92 |
| Cycle 3 | 4.42 | 5.46 | 2.54 | 5.35 | 6.41 |
| Cycle 4 | 4.13 | 1.92 | 3.91 | 3.84 | 6.06 |
| Cycle 5 | 0.86 | 0.00 | 0.00 | 1.47 | 0.42 |
| Cycle 6 | 2.08 | 4.61 | 0.97 | 2.56 | 3.35 |
| Cycle 7 | 2.66 | 8.44 | 1.17 | 3.72 | 3.46 |
| Cycle 8 | 3.46 | 0.00 | 3.76 | 6.81 | 0.00 |
| Cycle 9 | 6.98 | 2.52 | 8.02 | 8.77 | 3.58 |
| Cycle 10 | 2.84 | 2.32 | 0.00 | 8.28 | 0.00 |

# Treatment effects

Event probabilities were calculated by applying treatment effects to an estimate of baseline risk. In some instances, the best estimate of treatment effect was only available by synthesising evidence as described in the following sections.

## Ischaemic stroke

The ischaemic stroke (IS) risk in the model is conditional on the treatment administered, virtual CHADS_2_ score and AF status. The annual probabilities of IS events as implemented in the economic model were calculated using the network of evidence outlined below (Figure 1).

Figure 1. Network of evidence for risk of ischaemic stroke among patients with AF


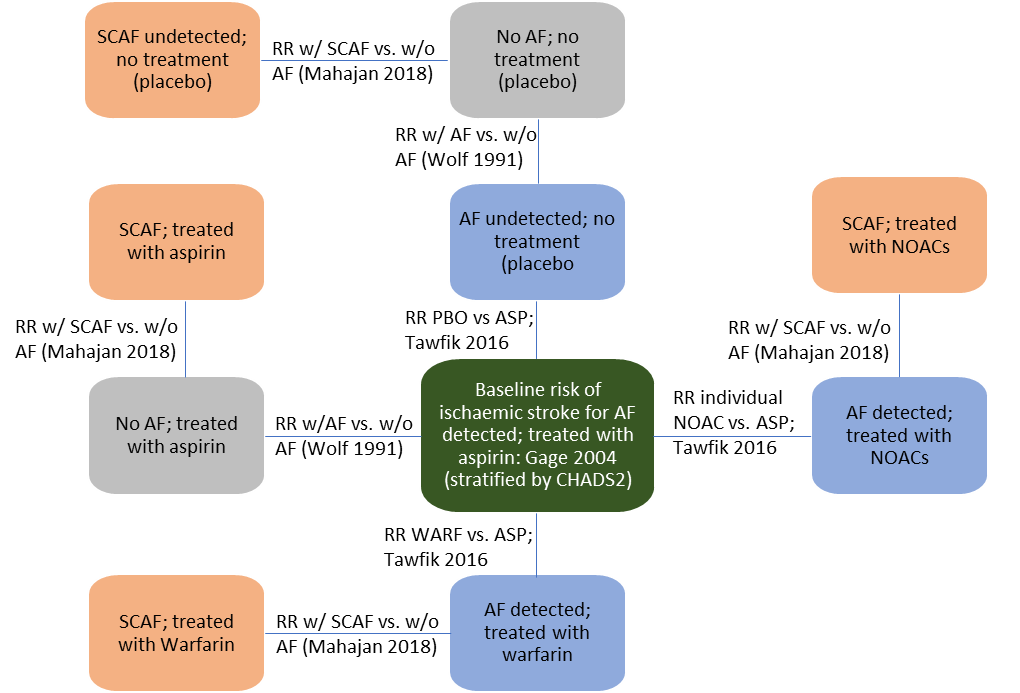


Gage et al. ([6](#_ENREF_6)) reported the annual risk of ischaemic stroke among patients with diagnosed AF receiving aspirin broken down by CHADS_2_ score. These data were used as baseline risk and synthesised with treatment effects to derive the risks associated with other treatments, e.g. non-vitamin K oral anticoagulant (NOAC) and warfarin and to derive the risks associated with being on aspirin treatment but not having AF.

In order to estimate the risk of recurrent IS in AF-free patients taking aspirin or no treatment, the risks reported by Gage et al.([6](#_ENREF_6)) were adjusted with a risk ratio (RR = 4.8) from Wolf 1991, to obtain the IS risk of patients without AF ([7](#_ENREF_7)).

In order to estimate the risk of recurrent IS in AF-free patients taking no treatment, warfarin or NOACs, the risks reported by Gage et al.([6](#_ENREF_6)) were synthesised with RR values from a recent systematic review and network meta-analysis of stroke prevention treatments in patients with AF ([8](#_ENREF_8)).

In order to estimate the risk of recurrent IS in patients with sub-clinical (or device detected) AF, a risk ratio (RR = 2.74 from Mahajan 2018 was applied to the IS risk estimated for AF-free patients ([9](#_ENREF_9)). The paper calculated this combined estimate of the RR, from the subgroup of studies include in their systematic review that reported CHADS_2_ score. These treatment effects were assumed to apply the heart failure subgroup also, in the absence of alternative evidence.

The annual IS risks by CHADS_2_ score are presented in Table 6, while the treatment effects for aspirin, warfarin and NOAC are presented in Table 7.

Table 6. Annual stroke risk (%)

| **CHADS_2_ score** | **Without AF** | | **With Subclinical AF** | | | |
| --- | --- | --- | --- | --- | --- | --- |
|  | **No treatment (placebo)** | **Aspirin** | **No treatment (placebo)** | **Aspirin** | **NOAC** | **Warfarin** |
| 0 | 0.2 | 0.2 | 0.6 | 0.4 | 0.2 | 0.1 |
| 1 | 0.6 | 0.5 | 1.6 | 1.1 | 0.4 | 0.4 |
| 2 | 1.3 | 0.9 | 3.2 | 2.3 | 0.9 | 0.8 |
| 3 | 2.5 | 1.8 | 6.2 | 4.4 | 1.7 | 1.6 |
| 4 | 3.2 | 2.3 | 7.9 | 5.6 | 2.1 | 2.1 |
| 5 | 3.6 | 2.6 | 9.0 | 6.3 | 2.4 | 2.3 |
| 6 | 4.0 | 2.9 | 10.1 | 7.1 | 2.7 | 2.6 |
| Source | ([7](#_ENREF_7)) | ([7](#_ENREF_7)) | ([8](#_ENREF_8), [9](#_ENREF_9)) | ([6](#_ENREF_6), [9](#_ENREF_9)) | ([8](#_ENREF_8), [9](#_ENREF_9)) | ([8](#_ENREF_8), [9](#_ENREF_9)) |

Abbreviations: AF, atrial fibrillation; NOAC, new oral anticoagulant

Table 7. Treatment effects for IS risks

| **Variable** | **Mean (95% CI)** | **Source** |
| --- | --- | --- |
| RR placebo vs aspirin | 1.40 (1.09 – 1.8) | ([8](#_ENREF_8)) |
| RR warfarin vs aspirin | 0.37 (0.29 – 0.48) | Reciprocal of RR value for aspirin vs warfarin ([8](#_ENREF_8)) |
| RR NOAC vs aspirin | 0.39 (0.28 – 0.54) | Average of all RR values of individual NOACs (APX, DBG 110, DBG 150, RVX, EDX HD, EDX LD) vs warfarin ([8](#_ENREF_8)) |

Abbreviations: APX, apixaban; CI, confidence interval; DBG, dabigatran; EDX HD, edoxaban high dose; EDX LD, edoxaban low dose; NOAC, new oral anticoagulant; RR, risk ratio; RVX, rivaroxaban

## Bleeding events

The risks and severity distribution of bleeds and hemorrhagic stroke for each type of treatment applied in the base-case analysis are summarised in Table 8.

Table 8. Annual probabilities and severity of bleeds and hemorrhagic stroke, by anticoagulant treatment received

|  | **No treatment** | **Aspirin** | **NOAC*****^,†^** | **Warfarin*** |
| --- | --- | --- | --- | --- |
|  |  |  |  |  |
| Intracranial hemorrhage (ICH)‡ | 0.2% | 0.5% | 0.3% | 0.8% |
| Hemorrhagic stroke (HS)§ | 0.1% | 0.3% | 0.2% | 0.5% |
| Other ICH | 0.1% | 0.2% | 0.1% | 0.3% |
| Extra-cranial hemorrhage (ECH)∥ | 2.0% | 2.5% | 2.7% | 3.1% |
| Gastrointestinal (GI) bleed | 0.8% | 1.0% | 1.1% | 1.3% |
| Other ECH | 1.2% | 1.4% | 1.6% | 1.8% |
| All major bleeds | 2.2% | 2.9% | 3.1% | 3.8% |
| CRNM bleed | 5.3% | 6.9% | 7.9% | 9.5% |
| **HS severity** | Mild: 28%; moderate: 23%; severe: 12% fatal: 37% ([10](#_ENREF_10)) | | | |
| **Case fatalities** | Due to other ICH: 13%, due to ECH: 2% ([10](#_ENREF_10)) | | | |

**Notes:** *, NOAC was used as treatment in base case, and warfarin was considered in sensitivity analysis; **^†^**, class-effect was assumed by taking the average across apixaban, dabigatran (low and high dose), rivaroxaban, edoxaban (low and high dose) (11); ‡, 59.7% of ICHs were assumed to be HS ([10](#_ENREF_10)); §, HS risk was adjusted by a factor of 1.97 (95% CI, 1.79-2.16) per decade ([11](#_ENREF_11)); ∥, 41.8% of ECHs were assumed to be GI bleeds ([10](#_ENREF_10)). These were updated for the heart failure subgroup, as detailed in the supplementary data.

**Abbreviations:** CRNM: clinically relevant non-minor bleed; ECH: extracranial hemorrhage; GI: gastrointestinal; HS: hemorrhagic stroke; ICH: intracranial hemorrhage; NOAC: new oral anticoagulant.

### Baseline risks

Similar to the IS risks, a set of probabilities was selected as baseline risk for one drug and treatment effects were applied to derive the risks for other drugs. For intracranial haemorrhagic (ICH), gastro-intestinal (GI) bleed and clinically relevant non-major (CRNM) bleed, the annual risk for patients on warfarin was estimated by averaging across the warfarin arms of studies that presented data for overall population with AF (ARISTOTLE, RELY, ROCKET and ENGAGE)([12-14](#_ENREF_12)). These values were used as the baseline risk of bleeding events (Table 9).

Table 9. Bleeding event risks with warfarin from each trial and pooled across trials

| **Population** | **Outcome** | **ARISTOTLE** | **RELY** | **ROCKET** | **ENGAGE** | **Mean warfarin risk** | **Source** |
| --- | --- | --- | --- | --- | --- | --- | --- |
| All patients | ICH | 0.80% | 0.74% | 0.70% | 0.85% | 0.78% | ([15-18](#_ENREF_15)) |
|  | GI Bleed | 0.79% | 1.02% | 2.16% | 1.23% | 1.28% | ([10](#_ENREF_10), [15](#_ENREF_15), [16](#_ENREF_16), [18](#_ENREF_18)) |
|  | CRNM Bleed | 3.00% | 16.37% | 11.40% | 10.15% | 9.51% | ([10](#_ENREF_10), [15](#_ENREF_15), [16](#_ENREF_16), [18](#_ENREF_18)) |
| Heart failure subgroup | ICH | 0.78% | 0.65% | 0.65% | 0.82% | 0.73% | ([19-22](#_ENREF_19)) |
|  | GI Bleed | Not reported | Not reported | Not reported | 1.28% | 1.28% | ([22](#_ENREF_22)) |
|  | CRNM Bleed | 3.00% | 16.37% | 11.40% | 10.15% | 9.51% | ([10](#_ENREF_10), [15](#_ENREF_15), [16](#_ENREF_16), [18](#_ENREF_18)) |

Abbreviations: CRNM, clinically relevant non-major; GI, gastro-intestinal; ICH, intra-cranial haemorrhage

### Treatment effects on bleeding events

The bleeding events risks in the model are conditional on the treatment administered. The annual probabilities of the bleeding events as implemented in the economic model were calculated using the network of evidence outlined below (Figure 2 for ICH, Figure 3 for major bleeds, Figure 4 for CRNM bleeds). Treatment effects in the heart failure subgroup are taken from published subgroup analyses of the same clinical trials (Table 11).

Figure 2. Network of evidence for ICH

Baseline risk of ICH for AF detected; treated with warfarin, average of WARF arms in NOAC trials (RELY, ROCKET, ARISTOTLE, ENGAGE)

Placebo

NOACs

Aspirin

NOAC vs Warfarin, average of (RELY, ROCKET, ARISTOTLE, ENGAGE)

RR ASP vs. WARF; Tawfik 2016

RR individual PBO vs. WARF; Tawfik 2016

Figure 3. Network of evidence for major bleeds

Baseline risk of major bleed for AF detected; treated with warfarin, average of WARF arms in NOAC trials (RELY, ROCKET, ARISTOTLE, ENGAGE)

Placebo

NOACs

Aspirin

NOAC vs Warfarin, average of (RELY, ROCKET, ARISTOTLE, ENGAGE)

RR ASP vs. WARF; Tawfik 2016

RR individual PBO vs. WARF; Tawfik 2016

Figure 4. Network of evidence for CRNM bleeds

Baseline risk of ICH for AF detected; treated with warfarin, average of WARF arms in NOAC trials (RELY, ROCKET, ARISTOTLE, ENGAGE)

Placebo

NOACs

Aspirin

RR PBO vs. WARF; CAFA

HR NOAC vs. ASP;

Connolly 2011

AVEROES

NOAC vs Warfarin, average of (RELY, ROCKET, ARISTOTLE, ENGAGE)

The published and derived treatment effects for all bleeding events are summarised in Table 10 and the resulting annual risk of such events are presented in Table 13.

Table 10. Treatment effects for bleeding events – all patients

| **Variable** | **Mean (95% CI)** | **Source** |
| --- | --- | --- |
| **ICH** |  | |
| RR placebo vs warfarin | 0.22 (0.07 – 0.65) | ([8](#_ENREF_8)) |
| RR aspirin vs warfarin | 0.64 (0.39 – 1.04) | ([8](#_ENREF_8)) |
| RR NOAC vs warfarin | 0.42 (0.19 – 0.91) | Average of all RR values of individual NOACs (APX, DBG 110, DBG 150, RVX, EDX HD, EDX LD) vs warfarin ([8](#_ENREF_8)) |
| **Major bleed** |  | |
| RR placebo vs warfarin | 0.57 (0.32 – 1) | ([8](#_ENREF_8)) |
| RR aspirin vs warfarin | 0.77 (0.61 – 0.98) | ([8](#_ENREF_8)) |
| RR NOAC vs warfarin | 0.80 (0.41 – 1.19) | Average of all RR values of individual NOACs (APX, DBG 110, DBG 150, RVX, EDX HD, EDX LD) vs warfarin ([8](#_ENREF_8)) |
| **CRNM bleed** |  | |
| RR placebo vs warfarin | 0.55 (0.32 – 0.97) | Reciprocal value of RR warfarin vs placebo ([23](#_ENREF_23)) |
| HR NOAC vs warfarin | 0.83 (0.66 – 1.04) | Average of all HR values of individual NOACs (APX, DBG 110, DBG 150, RVX, EDX HD, EDX LD) vs warfarin ([10](#_ENREF_10), [15](#_ENREF_15), [16](#_ENREF_16), [18](#_ENREF_18)) |
| HR NOAC vs aspirin | 1.15 (0.86 – 1.54) | ([24](#_ENREF_24)) |

Abbreviations: APX, apixaban; CI, confidence interval; CRNM, clinically relevant non-major; DBG, dabigatran; EDX HD, edoxaban high dose; EDX LD, edoxaban low dose; ICH, intra-cranial haemorrhage; HR, hazard ratio; NOAC, new oral anticoagulant; RR: risk ratio / relative risk; RVX, rivaroxaban

Table 11. Treatment effects for bleeding events – heart failure subgroup

| **Variable** | **Mean (95% CI)** | **Source** |
| --- | --- | --- |
| **ICH** |  | |
| RR placebo vs warfarin | 0.22 (0.07 – 0.65) | ([8](#_ENREF_8)) |
| RR aspirin vs warfarin | 0.64 (0.39 – 1.04) | ([8](#_ENREF_8)) |
| RR NOAC vs warfarin | 0.44 (0.14 – 1.02) | Average of all RR values of individual NOACs (APX, DBG 110, DBG 150, RVX, EDX HD, EDX LD) vs warfarin ([19-22](#_ENREF_19)) |
| **Major bleed** |  | |
| RR placebo vs warfarin | 0.57 (0.32 – 1) | ([8](#_ENREF_8)) |
| RR aspirin vs warfarin | 0.77 (0.61 – 0.98) | ([8](#_ENREF_8)) |
| RR NOAC vs warfarin | 0.84 (0.6 – 1.15) | RR value from ENGAGE (EDX HD, EDX LD) vs warfarin ([22](#_ENREF_22)) |
| **CRNM bleed** |  | |
| RR placebo vs warfarin | 0.55 (0.32 – 0.97) | Reciprocal value of RR warfarin vs placebo ([23](#_ENREF_23)) |
| HR NOAC vs warfarin | 0.87 (0.79 – 1.05) | Average of all HR values of individual NOACs (APX, DBG 110, DBG 150, RVX, EDX HD, EDX LD) vs warfarin ([10](#_ENREF_10), [15](#_ENREF_15), [16](#_ENREF_16), [18](#_ENREF_18)) |
| HR NOAC vs aspirin | 1.15 (0.86 – 1.54) | ([24](#_ENREF_24)) |

Abbreviations: APX, apixaban; CI, confidence interval; CRNM, clinically relevant non-major; DBG, dabigatran; EDX HD, edoxaban high dose; EDX LD, edoxaban low dose; ICH, intra-cranial haemorrhage; HR, hazard ratio; NOAC, new oral anticoagulant; RR: risk ratio / relative risk; RVX, rivaroxaban

# Event severity

## Ischaemic stroke severity

The proportion of ischaemic stroke events that are categorised as mild, moderate, severe or fatal was sourced from two published cost-effectiveness analyses ([10](#_ENREF_10), [25](#_ENREF_25)). The authors provided a distribution of severity for each drug separately; however, for the model presented here we assumed the severity distribution was not treatment-dependent. The average across all drugs, as well as the drug-specific distributions, are presented in Table 12.

Table 12. Ischaemic stroke severity distribution

| **Ischaemic stroke severity** | **APX** | **DBG (110)** | **DBG (150)** | **RVX** | **Aspirin** | **Warfarin** | **Overall** |
| --- | --- | --- | --- | --- | --- | --- | --- |
| % mild (mRS 0-2) | 53 | 35 | 35 | 49 | 36 | 45 | **42.2** |
| % moderate (mRS 3-4) | 21 | 28 | 22 | 18 | 38 | 30 | **26.2** |
| % severe (mRS 5) | 8 | 10 | 8 | 6 | 15 | 10 | **9.5** |
| % fatal (mRS 6) | 18 | 27 | 35 | 27 | 11 | 15 | **22.2** |

Abbreviations: APX, apixaban; DBG, dabigatran; EDX HD, edoxaban high dose; mRS, modified Rankin Scale; RVX, rivaroxaban

## Intracranial haemorrhage and extracranial haemorrhage

The proportion of ICH that are haemorrhagic strokes (HS) and the severity of those haemorrhagic strokes were reported for each drug in two published cost-effectiveness analyses ([16](#_ENREF_16), [19](#_ENREF_19)). For simplicity, we assumed the likelihood that an ICH was a haemorrhagic stroke was independent of treatment and thus took an average across all drugs (see Table 13). Similarly, the severity distribution was also assumed to be independent of treatment.

Extracranial haemorrhages are broken down into two events: GI bleeds and non-GI/non-ICH related bleeds (e.g. "other ECH"). Similarly to the ICH haemorrhages, we assumed the probability that an extracranial haemorrhage (ECH) was a GI bleeding event was independent of treatment and used an average from previously reported risks (Table 13)([10](#_ENREF_10), [25](#_ENREF_25)).

Table 13. ICH- and ECH-related probabilities

|  | **APX** | **DBG (110)** | **DBG (150)** | **RVX** | **Aspirin** | **Warfarin** | **Overall** |
| --- | --- | --- | --- | --- | --- | --- | --- |
| *ICH* | | | | | | | |
| %ICH are HS | 77 | 64 | 41 | 57 | 55 | 64 | **59.7** |
| % HS mild | 23 | 35 | 35 | 49 | 7 | 20 | **28.2** |
| % HS moderate | 32 | 28 | 22 | 18 | 20 | 15 | **22.5** |
| % HS severe | 10 | 10 | 8 | 6 | 27 | 12 | **12.2** |
| % HS fatal | 35 | 27 | 35 | 27 | 46 | 53 | **37.2** |
| % ICH are other ICH | 23 | 36 | 59 | 43 | 45 | 36 | **40.3** |
| % other ICH fatal |  | | | | | | **13.0** |
| *ECH* | | | | | | | |
| % ECH are GI bleed | 38 | 41 | 49 | 45 | 39 | 39 | **41.8** |
| % ECH fatal |  | | | | | | **2.0** |
| *Permanent OAC discontinuation following major bleeding event* | | | | | | | |
| % discontinue following HS | | | | | | | **100** |
| % discontinue following "other ICH" | | | | | | | **56** |
| % discontinue following ECH | | | | | | | **25** |

Abbreviations: APX, apixaban; DBG, dabigatran; ECH, extra-cranial haemorrhage; EDX HD, edoxaban high dose; HS, haemorrhagic stroke; ICH, intra-cranial haemorrhage; OAC, oral anticoagulant; RVX, rivaroxaban

# Mortality

## Non-cerebrovascular mortality

Age-dependant mortality was based on rates from the National Vital Statistics report ([26](#_ENREF_26)). Deaths from cerebrovascular events are explicitly modelled. To avoid double counting, the life-table risks were reduced by the proportion of cerebrovascular cases to the total all-cause cases (Table 14), to reflect a baseline mortality risk due any cause other than a cerebrovascular episode ([27](#_ENREF_27)). Since the baseline mortality risk and the adjustment factors are based on national data, the level of uncertainty in the model input was considered to be very low and did not warrant incorporation to the probabilistic sensitivity analysis (PSA).

Table 14. Non-cerebrovascular mortality as a percentage of all mortality

| **Deaths by age** | **45-54** | **55-64** | **65-74** | **75-84** | **85+** |
| --- | --- | --- | --- | --- | --- |
| Cerebrovascular diseases (per 100,000) | 12.3 | 30.3 | 76.4 | 263.1 | 993.5 |
| All-cause (per 100,000) | 401.5 | 885.8 | 1790.9 | 4472.6 | 13574 |
| Cerebrovascular death as % of all deaths | 3.1% | 3.4% | 4.3% | 5.9% | 7.3% |

Moreover, we considered that the model population includes patients with recent history of a non-fatal stroke or TIA, which, evidence shows, increases their risk of all-cause mortality ([28](#_ENREF_28)). Using data from Brønnum-Hansen et al. ([28](#_ENREF_28)) and Huybrechts et al. ([29](#_ENREF_29)) we estimated the hazard ratio of a non-fatal mild stroke on non-cerebrovascular death to be 1.97 compared to the general population.

## Mortality post recurrent stroke event

An excess mortality risk was applied to patients in post-stroke event health states; that is, after a recurrent stroke (Table 15). Brønnum-Hansen et al. ([28](#_ENREF_28)) reported a 2.71 mortality HR for patients after a non-fatal stroke versus the general population. We synthesised this with data from another study to adjust the HR for the severity of the stroke event.

Huybrechts et al. ([29](#_ENREF_29)) presented mortality HRs of patients experiencing events of different severity, expressed in modified Rankin Scale (mRS). Assuming that the HR from Brønnum-Hansen et al. ([28](#_ENREF_28)) relates to a mRS score of 2 in the Huybrechts et al.([29](#_ENREF_29)) data, we compared the HR of mRS 1 to 2 (average of 0 vs 1 and 1 vs 2) versus the mRS of 2 to derive a HR for mild stroke. A similar calculation was repeated for moderate stroke (3 to 4 vs 2), and severe stroke (5 vs 2).

Table 15. Estimation of excess all-cause mortality attributed to mild, moderate and severe stroke events

| **Risk factor** | **Hazard ratio** | **Notes / Source** |
| --- | --- | --- |
| Post non-fatal stroke (assumed mild) vs general population | 2.71 | ([28](#_ENREF_28)) |
| mRS 1 vs 0 | 1.18 | ([29](#_ENREF_29)) |
| mRS 2 vs 1 | 1.32 | ([29](#_ENREF_29)) |
| mRS 3 vs 2 | 1.16 | ([29](#_ENREF_29)) |
| mRS 4 vs 3 | 1.43 | ([29](#_ENREF_29)) |
| mRS 5 vs 4 | 2.23 | ([29](#_ENREF_29)) |
| **Re-categorised mRS factors from Huybrechts et al. (**[**29**](#_ENREF_29)**)** | | |
| mRS 1 to 2 vs 2 | 0.95 | =([1.18+1.32]/2)/1.32 |
| mRS 3 to 4 vs 2 | 1.71 | =1.32* ([1.16+1.43]/2) |
| mRS 5 vs 2 | 4.87 | =2.23*1.43*1.16*1.32 |
| **Adjusted values for model** | | |
| Mild stroke | 2.56 | =0.95*2.71 |
| Moderate stroke | 4.63 | =1.71*2.71 |
| Severe stroke | 13.18 | =4.87*2.71 |

## Treatment effects on all-cause mortality

Patients in the post-stroke health states continue to receive treatment with either placebo (no treatment), aspirin or OAC therapy. Randomised controlled trial evidence has shown these treatments to have an impact on all-cause mortality ([12-14](#_ENREF_12), [30-32](#_ENREF_30)).

The network of evidence available for all-cause mortality is presented in Figure 5, while treatment effects are listed in Table 16.

Figure 5. Network of evidence for all-cause mortality


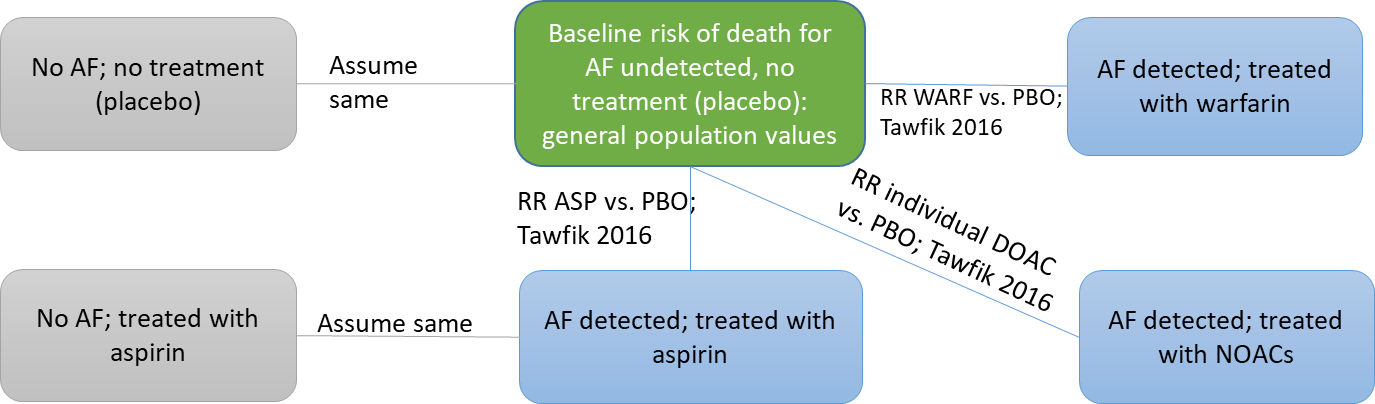


Table 16. Treatment effects for all-cause mortality

| **Variable** | **Mean (95% CI)** | **Source** |
| --- | --- | --- |
| RR aspirin vs placebo | 0.86 (0.69 – 1.06) | Reciprocal value of RR for placebo vs aspirin ([8](#_ENREF_8)) |
| RR warfarin vs aspirin | 0.82 (0.64 – 1.06) | ([8](#_ENREF_8)) |
| RR NOAC vs aspirin | 0.74 (0.56 – 0.98) | Average of all RR values of individual NOACs (APX, DBG 110, DBG 150, RVX, EDX HD, EDX LD) vs. PBO ([8](#_ENREF_8)) |

Abbreviations: APX, apixaban; DBG, dabigatran; EDX HD, edoxaban high dose; EDX LD, edoxaban low dose; RR, risk ratio; RVX, rivaroxaban

# Health-related quality of life

## Identifying utility values from the literature

Baseline utility of the population was taken from REVEAL – AF (data on file). Other health state utilities were sourced and synthesized from a range of published sources.

We performed a targeted literature review to identify health state utilities derived from a US population and/or using a US value set. The objective of the targeted review was to identify published cost-utility models evaluating interventions in patients with AF using a US payer perspective. From the identified studies, we extracted all available health state utility values and evaluated their goodness of fit to our model health states.

To identify relevant US cost-utility analyses in AF, we used the results of a 2015 systematic review of economic evaluations performed as part of the NICE Single Technology Appraisal of edoxaban ([33](#_ENREF_33)) and updated it with a pragmatic search in PubMed from 2015 to September 2018. Search terms included “atrial fibrillation” and “economic evaluation” or “cost effectiveness” or “cost utility” and were filtered for references published after January 1, 2015. Studies were included if they reported health state utility values used in a US economic evaluation of patients with either suspected or confirmed atrial fibrillation.

Twenty of the 52 full-text economic evaluations included in the edoxaban SLR used a US perspective and were screened alongside the 315 records identified through database searching. After reviewing titles and abstracts, 286 of the 335 records were excluded and 49 full-text articles were assessed against the eligibility criteria. Twenty-one articles were excluded after full-text review for reasons including having an unclear country perspective (n=9), not being an economic evaluation (n=6), not reporting any health state utility data (n=4) or providing insufficient information (n=2). Twenty-eight articles met the inclusion criteria and reported health state utility values were extracted and considered for inclusion in our economic model.

Twenty of the included economic evaluations used health states similar to those in our model, and all utility values in the publications could be linked back to a few key studies ([34-38](#_ENREF_34)). The methods in each original source were variable and not always clear. The most widely references sources were Gage 1996 and Sullivan 2005 ([35](#_ENREF_35), [37](#_ENREF_37)), which reported their methods and results in sufficient detail to inform health state utility estimates for stroke events and bleeds, respectively.

Some data points in the model could not be populated with US-specific utility values as they were not identified in the targeted literature review. This included data on the HRQoL impact of having AF and the added disutility of experiencing a recurrent stroke. For these data gaps, utility values from a UK cost-effectiveness analysis were used ([39](#_ENREF_39)). The values used in the UK analysis were derived from the OXVASC study for stroke-related health related quality of life (HRQoL)([40](#_ENREF_40)), which provided estimates of HRQoL over 5 years follow-up from an index stroke event using EQ-5D and UK population valuations.

The stroke utilities from Gage 1996 were assumed to apply during the cycle in which the acute stroke event occurred. Data from the OXVASC study showed that utility was likely to decrease slightly in the months and years following a mild stroke but improve following rehabilitation from a moderate or severe stroke. The relationship observed over time in the OXVASC study was used to derive post-event utilities from Gage 1996.

The disutility associated with acute events was confined to a certain duration. For strokes (ischemic or hemorrhagic) and other ICH, the acute disutility was assumed to last for the duration of one cycle (i.e. 3 months). For ECH, the acute disutility was assumed to last for 2 weeks and for CRNM bleeds, for just 2 days. The assumptions around the duration of these utility decrements were taken from publications by Dorian and Lip ([10](#_ENREF_10), [25](#_ENREF_25)). The authors arrived at these values through clinical expert opinion. Table 17 presents the utility scores used in the economic model for each health state and event.

**Table 17. Health state utilities**

| **Health state / event** | **Mean utility in study** | | **Source** |
| --- | --- | --- | --- |
|  | **All patients** | **Heart failure subgroup** |  |
| REVEAL AF baseline – All patients | 0.81 | 0.781 | REVEAL AF data on file |
| History of AF | 0.719 | 0.719 | OXVASC ([40](#_ENREF_40)) |
| Mild stroke event (IS or HS) | 0.755 | 0.732 | Gage 1996 ([35](#_ENREF_35)) |
| Moderate stroke event (IS or HS) | 0.390 | 0.376 | Gage 1996 ([35](#_ENREF_35)) |
| Severe stroke event (IS or HS) | 0.110 | 0.106 | Gage 1996 ([35](#_ENREF_35)) |
| Recurrent stroke event | 0.589 | 0.589 | OXVASC ([40](#_ENREF_40)) |
| Post mild stroke (IS or HS) | 0.755 | 0.729 | Gage 1996 ([35](#_ENREF_35)) & OXVASC ([40](#_ENREF_40)) |
| Post moderate stroke (IS or HS) | 0.454 | 0.437 | Gage 1996 ([35](#_ENREF_35)) & OXVASC ([40](#_ENREF_40)) |
| Post severe stroke (IS or HS) | 0.336 | 0.324 | Gage 1996 ([35](#_ENREF_35)) & OXVASC ([40](#_ENREF_40)) |
| Post recurrent stroke | 0.659 | 0.659 | OXVASC ([40](#_ENREF_40)) |
| Other ICH event | 0.819 | 0.000 | Dorian 2013; ([25](#_ENREF_25)) Lip 2014 ([10](#_ENREF_10)) from Sullivan 2011 ([41](#_ENREF_41)) |
| CRNM bleed | 0.999^*^ | 0.999 | ([10](#_ENREF_10), [25](#_ENREF_25), [41](#_ENREF_41)) |
| ECH | 0.993^**^ | 0.993 | ([10](#_ENREF_10), [25](#_ENREF_25), [41](#_ENREF_41)) |
| ^*^ disutility from CRNM bleed assumed to last 2 days  ^**^ disutility from ECH assumed to last 2 weeks | | | |

## Synthesis of utility values

The baseline utility value (U_Base_)([42](#_ENREF_42)) for the starting population was based on REVEAL AF and was adjusted by 0.00029 each year to reflect the natural decline of patients’ physical and mental functions due to age and other comorbidities. The health state utilities in Table 17 were synthesized with this baseline utility using multipliers (φ) derived by comparing the observed values from the literature against the utility expected for a similarly aged general population ([43](#_ENREF_43))(Table 18). The final utility used in the model was UBase* φHS.

**Table 18. Health state and acute event utility multipliers**

| **Health state** | **Multipliers (φ)** | |
| --- | --- | --- |
|  | **All patients** | **Heart failure subgroup** |
| No AF | 1 | 1 |
| AF | 0.9809 | 0.9809 |
| Post-mild stroke - No AF | 0.9172 | 0.9225 |
| Post-mild stroke - AF | 0.8997 | 0.9049 |
| Post-moderate stroke - No AF | 0.5502 | 0.5533 |
| Post-moderate stroke - AF | 0.5396 | 0.5428 |
| Post-severe stroke - No AF | 0.4071 | 0.4095 |
| Post-severe stroke - AF | 0.3994 | 0.4017 |
| Dead |  |  |
| Acute event |  |  |
| Mild recurrent stroke | 0.8996 | 0.9131 |
| Moderate recurrent stroke | 0.4616 | 0.4686 |
| Severe recurrent stroke | 0.1302 | 0.1322 |
| other ICH | 0.8190 | 0.8190 |
| ECH | 0.9931 | 0.9931 |
| CRNM bleed | 0.9990 | 0.9990 |

# Resource use and costs

## Cost sources

In this study, 2020 costs were used throughout. Where costs were not available from 2020, costs were inflated using the Medical Care component of the consumer price index ([44](#_ENREF_44)).

## Conventional follow-up (SoC) resource use

The cost of standard of care monitoring was informed by real-world administrative claims data on the monitoring accessed by this patient population ([45](#_ENREF_45)). On average, this patient group received 0.17 24-hour Holter monitors per year and 3.4 ECG monitors per year. The estimated unit costs for a 24-hour Holter monitor ($92.59, CPT 93224) and for an ECG ($25, CPT 93000) were sourced from Medicare national average payment amounts.

In order to simulate the diagnostic efficacy of SoC, clinical experts (co-authors KW, ME, MR) were asked to describe the conventional follow-up or SoC for patients deemed at high risk of AF. There was consensus to assume one 24-hr Holter monitoring each year to model diagnostic efficacy, leading to a relative accuracy of Reveal vs. SoC of 33.9 ([3](#_ENREF_3)).

## ICM resource use

Follow-up for ICM patients was informed by the REVEAL trial, using the REVEAL LINQ Registry; patients received a combination of remote and in person monitoring ([46](#_ENREF_46)).

## Drug and INR monitoring costs

Patients treated with warfarin accrued costs associated with international normalized ratio (INR) monitoring. This included an annual $18.80 laboratory test and $81.32 office visit, both sourced from Medicare national average payment amounts.

The cost per cycle for each drug and INR monitoring is displayed in Table 19.

Table 19. Drug and monitoring costs

| **Drug** | **Cost per cycle** | **Source** |
| --- | --- | --- |
| Aspirin | $54.16 | ([45](#_ENREF_45))[32] |
| Warfarin | $17.71 |  |
| Quarterly Warfarin INR monitoring | $24.60 | Medicare national average payment amounts |
| NOAC | $118.73 | Average cost of daily:  300 mg of Dabigatran ($99.11)  220 mg of Dabigatran ($97.24)  20 mg of Rivaroxaban ($103.26)  10 mg of Apixaban ($140.88)  60 mg of Edoxaban (30 mg pill) ($57.83)  60 mg of Edoxaban (60 mg pill) ($83.79)  ([45](#_ENREF_45)) |

## Acute care costs associated with clinical events

Stroke costs were categorised according to type (i.e. haemorrhagic or non-haemorrhagic), severity (mild, moderate, severe or fatal) and time (acute or post-acute maintenance). Costs for each permutation of these categories were sourced from Miller 2016 ([47](#_ENREF_47)). The same applies to the costs of major bleeds (other ICH, GI bleed, other ECH) and CRNM bleeds.

To model uncertainty around these costs, it was assumed that +/- 30% represented the interquartile range, due to the absence of any empirical data. The same is true for implantation, explantation and monitoring costs.

# Probabilistic sensitivity analysis

The parameters used within probabilistic sensitivity analysis are reported in Table 20. A lognormal distribution was used to vary costs and treatment effects (e.g. risk ratios and hazard ratios), as these are recommended for parameters that are positively skewed and bounded by zero. A Dirichlet distribution was used to vary the distribution of event severity, as this distribution is recommended for categorical inputs that sum to one. The beta distribution was used to vary risks, health state utility values and disutilities associated with health events. Other inputs were not varied within the probabilistic sensitivity analysis.

Table 20. Parameters included within probabilistic sensitivity analysis

| **Parameter** | **Mean** | **Confidence interval** | **Distribution** |
| --- | --- | --- | --- |
| **Probability of detection** | | | |
| Proportion of true AF detected by Reveal | 0.96 | (0.92, 1.00) | beta |
| **HR – reveal vs SoC** |  |  |  |
| Base Case | 33.88 | (13.24, 54.51) | Lognormal |
| CHADS_2_ 2 | 39000000 |  |  |
| CHADS_2_ 3 | 4.89 | (1.41, 16.90) | Lognormal |
| CHADS_2_ 4 + | 8.49 | (1.97, 36.50) | Lognormal |
| Base case (scenario analysis) | 813.00 |  | No sampling |
| **AF detection (>=6 min events)** | | | |
| Base-case detection |  |  |  |
| Detected AF at month 30 - Reveal-AF | 0.40 | (0.33, 0.46) | Beta |
| Detected AF at month 3 - Reveal-AF | 0.14 | (0.10, 0.17) | Beta |
| CHADS_2_ 2 detection |  |  |  |
| Detected AF at month 30 - Reveal-AF | 0.34 |  | No sampling |
| Detected AF at month 3 - Reveal-AF | 0.13 |  | No sampling |
| CHADS_2_ 3 detection |  |  |  |
| Detected AF at month 30 - Reveal-AF | 0.49 |  | No sampling |
| Detected AF at month 3 - Reveal-AF | 0.16 |  | No sampling |
| CHADS_2_ 4+ detection |  |  |  |
| Detected AF at month 30 - Reveal-AF | 0.37 |  | No sampling |
| Detected AF at month 3 - Reveal-AF | 0.13 |  | No sampling |
| **AF detection (>=5.5 hrs events)** | | | |
| Base-case detection |  |  |  |
| Detected AF at month 30 - Reveal-AF | 0.20 | (0.15, 0.25) | Beta |
| Detected AF at month 3 - Reveal-AF | 0.07 | (0.05, 0.10) | Beta |
| CHADS_2_ 2 detection |  |  |  |
| Detected AF at month 30 - Reveal-AF | 0.16 |  | No sampling |
| Detected AF at month 3 - Reveal-AF | 0.08 |  | No sampling |
| CHADS_2_ 3 detection |  |  |  |
| Detected AF at month 30 - Reveal-AF | 0.20 |  | No sampling |
| Detected AF at month 3 - Reveal-AF | 0.08 |  | No sampling |
| CHADS_2_ 4+ detection |  |  |  |
| Detected AF at month 30 - Reveal-AF | 0.24 |  | No sampling |
| Detected AF at month 3 - Reveal-AF | 0.06 |  | No sampling |
| **AF detection (51.5 months)** | | | |
| Base-case detection |  |  |  |
| Detected AF at month 51 | 0.55 |  | No sampling |
| Duration |  |  |  |
| Device longevity (years) | 3.00 |  |  |
| Unplanned explanatory per cycle | 0.01 |  | No sampling |
| infection – first cycle | 0.01 | (0.00, 0.01) | Beta |
| **Risk of ischaemic stroke** | | | |
| **Aspirin** |  |  |  |
| CHADS_2_ 0 | 0.01 | (0.00, 0.02) | Beta |
| CHADS_2_ 1 | 0.02 | (0.02, 0.03) | Beta |
| CHADS_2_ 2 | 0.05 | (0.04, 0.06) | Beta |
| CHADS_2_ 3 | 0.09 | (0.07, 0.11) | Beta |
| CHADS_2_ 4 | 0.11 | (0.08, 0.15) | Beta |
| CHADS_2_ 5 | 0.12 | (0.07, 0.23) | Beta |
| CHADS_2_ 6 | 0.14 | (0.02, 0.97) | Beta |
| **Treatment Effects** |  |  |  |
| Rate ratio NOAC (class effect) vs aspirin | 0.39 | (0.28, 0.54) | Lognormal |
| **Risk adjustments** | | | |
| Risk ratio with AF vs without AF | 4.80 | (3.36, 6.24) | Lognormal |
| Risk ratio with SCAF vs without AF | 2.40 | (1.46, 5.23) | Lognormal |
| Risk ratio for stroke risk per decade age | 1.46 | (0.80, 2.16) | Lognormal |
| **Ischaemic stroke severity** | | | |
| Mild | 0.42 |  | Dirichlet |
| Moderate | 0.26 |  | Dirichlet |
| Severe | 0.10 |  | Dirichlet |
| Fatal | 0.22 |  | Dirichlet |
| **Bleeding risks** | | | |
| RR for bleeding risk per decade age | 1.97 | (1.79, 2.16) | Lognormal |
| **Major Bleeding** | | | |
| Rate ratio NOAC (class effect) vs warfarin | 0.80 | (0.41, 1.19) | Lognormal |
| Rate ratio aspirin vs warfarin | 0.77 | (0.61, 0.98) | Lognormal |
| Rate ratio placebo vs warfarin | 0.57 | (0.32, 1.00) | Lognormal |
| **ICH** | | | |
| Annual risk - warfarin | 0.01 | (0.01, 0.01) | Beta |
| Rate ratio NOAC (class effect) vs warfarin | 0.42 | (0.19, 0.91) | Lognormal |
| Rate ratio aspirin vs warfarin | 0.64 | (0.39, 1.04) | Lognormal |
| Rate ratio placebo vs warfarin | 0.22 | (0.07, 0.65) | Lognormal |
| Proportion of ICH that are Haemorrhagic Strokes | 0.60 |  | No sampling |
| **Haemorrhagic stroke severity** | | | |
| Mild | 0.28 |  | Dirichlet |
| Moderate | 0.23 |  | Dirichlet |
| Severe | 0.12 |  | Dirichlet |
| Fatal | 0.37 |  | Dirichlet |
| **GI Bleed** | | | |
| Annual risk - warfarin | 0.01 | (0.01, 0.02) | Beta |
| Proportion of ECH that are GI bleeds | 0.42 |  | No sampling |
| **CRNM Bleed** | | | |
| Annual risk - warfarin | 0.10 | (0.03, 0.16) | Beta |
| HR NOAC (class effect) vs warfarin | 0.83 | (0.66, 1.04) | Lognormal |
| Rate ratio warfarin vs placebo | 1.80 | (1.03, 3.15) | Lognormal |
| HR NOAC (class effect) vs aspirin | 1.15 | (0.86, 1.54) | Lognormal |
| **Discontinuation** | | | |
| % discontinue after “other ICH” | 0.56 | (0.43, 0.69) | Beta |
| % discontinue after ECH | 0.25 | (0.01, 0.69) | Beta |
| % discontinue annually, other reasons |  |  |  |
| Warfarin | 0.27 | (0.17, 0.41) | Beta |
| NOAC | 0.15 | (0.10, 0.23) | Beta |
| **Mortality** | | | |
| **Case fatality** |  |  |  |
| Other ICH | 0.13 | (0.06, 0.22) | Beta |
| Major bleed | 0.02 | (0.01, 0.03) | Beta |
| **Non-cerebrovascular mortality** | | | |
| HR post-mild stroke vs general population | 1.97 |  | No sampling |
| HR no stroke | 1.00 |  | No sampling |
| **All-cause** |  |  |  |
| HR post-mild stroke vs general population | 2.56 |  | No sampling |
| HR post-moderate stroke vs general population | 4.63 |  | No sampling |
| HR post-severe stroke vs general population | 13.19 |  | No sampling |
| Rate ratio placebo vs. aspirin | 1.16 | (0.94, 1.44) | Lognormal |
| Rate ratio placebo vs. warfarin | 1.22 | (0.94, 1.57) | Lognormal |
| Rate ratio NOAC (class effect) vs placebo | 0.74 | (0.56, 0.98) | Lognormal |
| **Costs** | | | |
| **Detection** |  |  |  |
| Reveal device & implantation | 6402.00 | (4481.40, 8322.60) | Lognormal |
| Reveal explantation | 671.00 | (469.70, 872.30) | Lognormal |
| Reveal interrogation |  |  |  |
| Cost per in-person interrogation | 36.00 |  | No sampling |
| Cost per remote interrogation | 64.00 |  | No sampling |
| Frequency of in-person monitoring (per cycle) | 0.22 |  | No sampling |
| Frequency of remote monitoring (per cycle) | 1.56 |  | No sampling |
| Unscheduled visits |  |  |  |
| Frequency (per cycle) | 0.00 |  | No sampling |
| Cost per unscheduled visit | 81.32 |  | No sampling |
| Reveal diagnosis | 39.60 |  | No sampling |
| Unit cost of ECG | 25.00 | (17.50, 32.50) | Lognormal |
| Unit cost of Holter and event recorder (24-hour Holter) | 92.59 | (64.81, 120.37) | Lognormal |
| **Treatment monitoring** |  |  |  |
| INR monitoring (annual) – warfarin only | 98.41 |  | no sampling |
| **Acute events 3-month cost** |  |  |  |
| Mild Ischaemic stroke | 21,567 | (15,907, 28,037) | Lognormal |
| Moderate Ischaemic stroke | 25,124 | (17,587, 32,662) | Lognormal |
| Severe Ischaemic stroke | 32,279 | (22,596, 41,963) | Lognormal |
| Fatal ischaemic stroke | 32,379 | (22,596, 41,963) | Lognormal |
| Mild Haemorrhagic stroke | 23,622 | (16,536, 30,709) | Lognormal |
| Moderate Haemorrhagic stroke | 33,40 | (23,386, 43,431) | Lognormal |
| Severe Haemorrhagic stroke | 43,195 | (30,236, 56,153) | Lognormal |
| Fatal haemorrhagic stroke | 43,195 | (30,236, 56,153) | Lognormal |
| Other ICH | 25,149 | (17,604, 32,693) | Lognormal |
| GI bleed | 9.136 | (6,395, 11,877) | Lognormal |
| Other major ECH | 13,813 | (9,669, 17,957) | Lognormal |
| CRNM bleed | 1,163 | (2,267, 4,211) | Lognormal |
| **Post-event annual costs** |  |  |  |
| Post-mild stroke (IS or HS) | 3,239 | (2,267, 4,211) | Lognormal |
| Post-moderate stroke (IS or HS) | 8,845 | (6,192, 11,499) | Lognormal |
| Post-severe stroke (IS or HS) | 19,212 | (13,448, 24,975) | Lognormal |
| **Utilities** | | | |
| Baseline | 0.81 | (0.71, 0.91) | Beta |
| **Acute events** |  |  |  |
| Mild stroke (IS or HS) | 0.76 | (0.66, 0.86) | Beta |
| Moderate stroke (IS or HS) | 0.39 | (0.35, 0.43) | Beta |
| Severe stroke (IS or HS) | 0.11 | (0.09, 0.13) | Beta |
| **Post-event** |  |  |  |
| Post-mild stroke (IS or HS) | 0.76 | (0.74, 0.78) | Beta |
| Post-moderate stroke (IS or HS) | 0.45 | (0.38, 0.52) | Beta |
| Post-severe stroke (IS or HS) | 0.34 | (0.21, 0.47) | Beta |
| **Disutilities** |  |  |  |
| History of AF | -0.014 | (-0.051, 0.024) | Normal |
| Recurrent stroke event (IS or HS) | -0.150 | (-0.228, -0.073) | Normal |
| Post recurrent stroke (IS or HS) | -0.068 | (-0.118, -0.023) | Normal |
| CRNM bleed | -0.181 | (-0.208, -0.154) | Normal |
| ECH | -0.181 | (-0.208, -0.154) | Normal |
| Other ICH | -0.181 | (-0.08, -0.154) | Normal |
| Decrement for age | -0.00029 | (-0.0003, -0.00025) | Normal |
| **Duration of disutility (years)** |  |  |  |
| CRNM bleed | 0.00548 |  | No sampling |
| ECH | 0.0384 |  | No sampling |

**Abbreviations:** AF: atrial fibrillation, CRNM: clinically relevant non-major, ECH: extracranial hemorrhage, GI: gastrointestinal, HR: hazard ratio, HS: hemorrhagic stroke, ICER: incremental cost-effectiveness ratio. ICH: intracranial hemorrhage, ICM: insertable cardiac monitor, INR: International Normalized Monitoring, IS: ischemic stroke, NOAC: non-vitamin K oral anticoagulants, QALY: quality-adjusted life-year, SoC: standard of care.

# Supplementary File References

1. Reiffel, J.A., et al., JAMA Cardiol, 2017. **2**(10): p. 1120-1127.

2. Glotzer, T.V., et al., Circ Arrhythm Electrophysiol, 2009. **2**(5): p. 474-80.

3. Reiffel JA, V.A., Kowey P, Halperin J, Gersh B, Elkind MSV, et al, Journal of the American College of Cardiology, 2018. **71**(11 Supplement): p. A274.

4. Sanna, T., et al., N Engl J Med, 2014. **370**(26): p. 2478-86.

5. Diamantopoulos, A., et al., Int J Stroke, 2016. **11**(3): p. 302-12.

6. Gage, B.F., et al., Circulation, 2004. **110**(16): p. 2287-92.

7. Wolf, P.A., R.D. Abbott, and W.B. Kannel, Stroke, 1991. **22**(8): p. 983-8.

8. Tawfik, A., et al., Clin Pharmacol, 2016. **8**: p. 93-107.

9. Mahajan, R., et al., 2018(1522-9645 (Electronic)).

10. Lip, G.Y., et al., Clin Ther, 2014. **36**(2): p. 192-210 e20.

11. Nasir, J.M., et al., Heart Rhythm, 2017. **14**(7): p. 955-961.

12. Easton, J.D., et al., Lancet Neurol, 2012. **11**(6): p. 503-11.

13. Diener, H.C., et al., Lancet Neurol, 2012. **11**(3): p. 225-31.

14. Hankey, G.J., et al., Lancet Neurol, 2012. **11**(4): p. 315-22.

15. Connolly, S.J., et al., N Engl J Med, 2009. **361**(12): p. 1139-51.

16. Giugliano, R.P., et al., N Engl J Med, 2013. **369**(22): p. 2093-104.

17. Granger, C.B., et al., N Engl J Med, 2011. **365**(11): p. 981-92.

18. Patel, M.R., et al., N Engl J Med, 2011. **365**(10): p. 883-91.

19. van Diepen, S., et al., Circ Heart Fail, 2013. **6**(4): p. 740-7.

20. Ferreira, J., et al., Eur J Heart Fail, 2013. **15**(9): p. 1053-61.

21. Avezum, A., et al., Circulation, 2015. **132**(8): p. 624-32.

22. Magnani, G., et al., European Journal of Heart Failure, 2016. **18**(9): p. 1153-1161.

23. Connolly, S.J., et al., J Am Coll Cardiol, 1991. **18**(2): p. 349-55.

24. Connolly, S.J., et al., N Engl J Med, 2011. **364**(9): p. 806-17.

25. Dorian, P., et al., Eur Heart J, 2014. **35**(28): p. 1897-906.

26. Office for National Statistics 2017.

27. Office for National Statistics 2014.

28. Bronnum-Hansen, H., et al., Stroke, 2001. **32**(9): p. 2131-6.

29. Huybrechts, K.F., et al., Cerebrovasc Dis, 2008. **26**(4): p. 381-7.

30. Diener, H.C., et al., Lancet Neurol, 2010. **9**(12): p. 1157-63.

31. Ntaios, G., et al., Stroke, 2012. **43**(12): p. 3298-304.

32. Lancet, 1993. **342**(8882): p. 1255-62.

33. National Institute for Health and Care Excellence. 2013: London.

34. Fryback, D.G., et al., Medical Decision Making, 1993. **13**(2): p. 89-102.

35. Gage, B.F., A.B. Cardinalli, and D.K. Owens, Arch Intern Med, 1996. **156**(16): p. 1829-36.

36. O'Brien, C.L. and B.F. Gage, Jama, 2005. **293**(6): p. 699-706.

37. Sullivan, P.W., W.F. Lawrence, and V. Ghushchyan, Med Care, 2005. **43**(7): p. 736-49.

38. Thomson, R., et al., Lancet, 2000. **355**(9208): p. 956-62.

39. Diamantopoulos, A., et al., International Journal of Stroke, 2016. **11**(3): p. 302-312.

40. Luengo-Fernandez, R., et al., Neurology, 2013. **81**(18): p. 1588-95.

41. Sullivan, P.W., et al., Med Decis Making, 2011. **31**(6): p. 800-4.

42. Sullivan, P.W. and V. Ghushchyan, Med Decis Making, 2006. **26**(4): p. 410-20.

43. Ara, R. and J.E. Brazier, Value Health, 2010. **13**(5): p. 509-18.

44. Federal Reserve Bank of St. Louis. 2020; Available from: <https://fred.stlouisfed.org/series/CPIMEDSL#0>.

45. Optum, Electronic Health Record de-identified database 2007-2017.

46. Medtronic Cardiac Rhythm and Heart Failure. Reveal LINQ Registry. 2019 [cited 2019; Available from: <https://clinicaltrials.gov/ct2/show/NCT02746471>.

47. Miller, J.D., et al., 2016(1178-6981 (Print)).
